# Colorless Near‐Infrared Absorbing Dyes Based on B‐N Fused Donor‐Acceptor‐Donor π‐Conjugated Molecules for Organic Phototransistors

**DOI:** 10.1002/advs.202405656

**Published:** 2024-06-14

**Authors:** Soichi Yokoyama, Sakura Utsunomiya, Takuji Seo, Akinori Saeki, Yutaka Ie

**Affiliations:** ^1^ The Institute of Scientific and Industrial Research (SANKEN) Osaka University 8‐1 Mihogaoka Ibaraki Osaka 567‐0047 Japan; ^2^ Innovative Catalysis Science Division Institute for Open and Transdisciplinary Research Initiatives (OTRI) Osaka University 2‐1 Yamadaoka Suita Osaka 565‐0871 Japan; ^3^ Department of Applied Chemistry Graduate School of Engineering Osaka University 2‐1 Yamadaoka Suita Osaka 565‐0871 Japan

**Keywords:** colorless characteristics, electronic transitions, NIR absorbing dyes, organic phototransistors, organic semiconductors

## Abstract

The introduction of a colorless function to organic electronic devices allows responses to light in the near‐infrared (NIR) region and is expected to broaden the applications of these devices. However, the development of a colorless NIR dye remains a challenge due to the lack of a rational molecular design for controlling electronic transitions. In this study, to suppress the π‐π* transitions in the visible region, polycyclic donor‐acceptor‐donor π‐conjugated molecules with boron bridges (**Py‐FNTz‐B** and **IP‐FNTz‐B**) are designed and synthesized, which contain pyrrole or indenopyrrole as donor units with fluorinated naphthobisthiadiazole (FNTz) as an acceptor unit. The pyrrole end‐capped **Py‐FNTz‐B** shows an absorption band in the NIR region without distinct visible‐light absorption, which has led to the establishment of colorless characteristics. The indenopyrrole end‐capped **IP‐FNTz‐B** shows a narrow optical energy gap of 0.87 eV in films. Time‐resolved microwave conductance and field‐effect transistors demonstrate the semiconducting characteristics of these molecules, and **Py‐FNTz‐B**‐based devices function as NIR phototransistors. Theoretical analyses indicate that the combination of a polyene‐like electronic structure with orbital symmetry is important to obtain NIR wavelength‐selective absorption. This study suggests that a molecular design based on electronic structures can be effective in the development of colorless NIR‐absorbing dyes for organic electronics.

## Graphical abstract

To develop a colorless near‐infrared (NIR) absorbing dye for organic electronics, polycyclic donor‐acceptor‐donor types of π‐conjugated molecules with boron bridges are synthesized. These molecules show NIR wavelength‐selective absorption, which leads to colorless characteristics. Herein, a rational molecular design is proposed based on theoretical analyses. Semiconducting characteristics including NIR‐responsible phototransistors are also described.

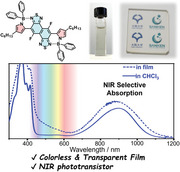

## 1 Introduction

The development of high‐performance organic semiconducting materials has enabled the embodiment of organic thin‐film electronics such as organic light‐emitting diodes, organic solar cells, and organic field‐effect transistors (OFETs).^[^ ^1^ ^]^ As one of the next logical applications, organic electronic devices that could respond to light in the near‐infrared (NIR) region would be in high demand due to distinct advantages in photodetection, bioimaging, chemotherapy, optical filters, and security marking.^[^ ^2^ ^]^ To realize such NIR‐type devices, it is essential to develop novel organic semiconductors with a bandgap of less than 1.5 eV (equivalent to wavelengths longer than 800 nm) as well as charge‐transport characteristics. Narrow bandgaps can be produced by using one of the donor (D)‐acceptor (A) configurations,^[^ ^3^ ^]^ quinoidal conjugations,^[^ ^4^ ^]^ phthalocyanines,^[^ ^5^ ^]^ polyrylenes,^[^ ^6^ ^]^ cyanine dyes,^[^ ^7^ ^]^ antiaromatic conjugations,^[^ ^8^ ^]^ radical characteristics,^[^ ^9^ ^]^ and B‐N fused structures.^[^ ^10^ ^]^ However, precedents whereby these compounds also possess semiconducting characteristics have been still limited.^[^ ^3^, ^4^, ^7^ ^]^

Unlike the visible region, the NIR region is invisible, and the introduction of an additional function such as “colorless and transparent” has resulted in the development of novel NIR‐response organic electronic devices.^[^ ^3^, ^7^ ^]^ Although several NIR absorption dyes are known to furnish colorless characteristics,^[^ ^6^, ^9^, ^10^ ^]^ the establishment of a rational design principle for molecular structure and application to electronic devices remains a challenge that has not been developed.

**Figure 1:**
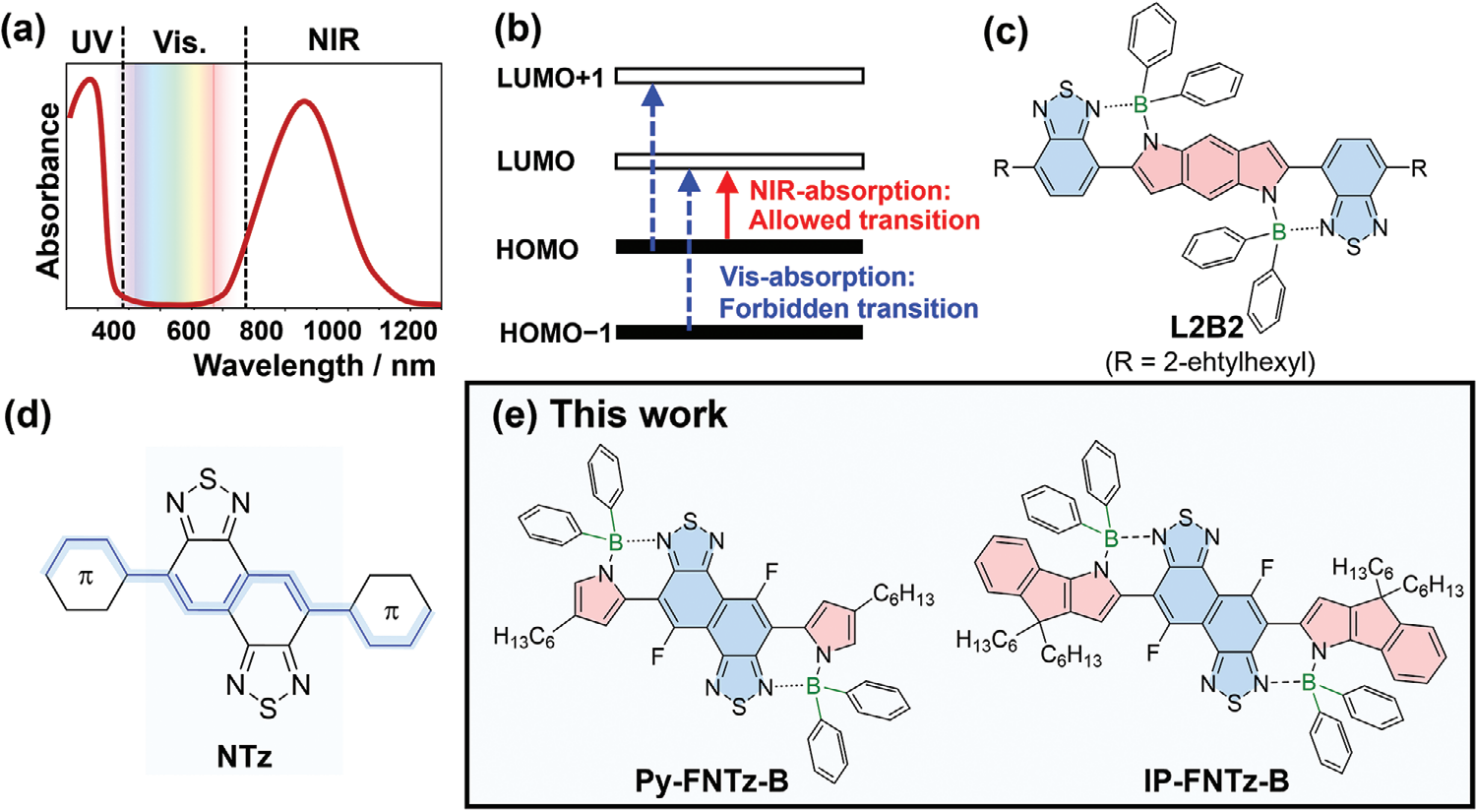
a) Target absorption spectrum and b) electronic transitions for colorless NIR dyes developed in this study. c) Molecular structure of reference compound **L2B2**. d) Polyene‐like structure of an NTz‐based π‐conjugated molecule. e) Molecular structures of **Py‐FNTz‐B**, **IP‐FNTz‐B**, and **IP‐FNTz‐B**.

Obtaining colorless characteristics for NIR absorption dyes based on π‐extended molecules requires 2 developments: 1) Narrowing the absorption bandgap to reach the NIR region; and, 2) Suppressing electronic transitions that correspond to the absorption of visible light (**Figure** **1**a,b). Nakamura et al. reported that the **L2B2** molecule, which is composed of A‐D‐A types of structures with B‐N linkages, could achieve an absorption spectrum that reaches 1100 nm (Figure 1c).^[^ ^10e^ ^]^

However, **L2B2** projects a blue color when used in a solution due to the transitions that are allowed in the visible region. We hypothesized that a colorless NIR absorption dye could be achieved via a molecular design wherein the first excited state shows an allowed transition while the second and third excited states are forbidden transitions (Figure 1b). Focusing on the electronic structure of **L2B2**, the central benzodipyrrole unit would cause multiple π‐π* transitions due to the orbital symmetries of the benzene ring, which would result in broad absorption bands in the vis‐NIR region. We assumed that tuning the electronic structures could accomplish NIR wavelength‐selective absorption and that the contribution of the polyene‐like electronic structure of naphthobisthiadiazole (NTz) would suppress multiple π‐π* transitions via a resonance effect (Figure 1d). In addition, the strong electron‐accepting nature of the NTz unit has the advantage of acquiring a narrow bandgap within a D‐A system. In this context, we developed a fluorinated NTz, defined as FNTz, as a strong electron‐accepting unit that contains D‐A type derivatives with high carrier‐transport characteristics.^[^ ^11^ ^]^ Therefore, in this study, we designed and synthesized polycyclic D‐A‐D type π‐conjugated molecules with boron bridges between the D and A units (**Py‐FNTz‐B**, **IP‐FNTz‐B**), as shown in Figure 1e. The pyrrole‐capped **Py‐FNTz‐B** showed maximum absorption at 900 nm and colorless and transparent characteristics in thin films. **IP‐FNTz‐B** has terminal D units that are composed of indenopyrrole and show a maximum absorption wavelength of 1200 nm with an onset that reaches upward of 1500 nm. Furthermore, these molecules exhibited semiconductor characteristics in OFETs, and **Py‐FNTz‐B** has also been used to produce organic phototransistors (OPTs) with current amplification in response to NIR light.

## 2 Results and Discussion

### 2.1 Computational Studies

**Scheme 1:**
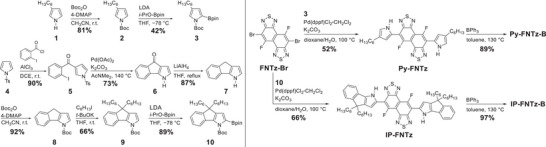
Synthetic routes for **Py‐FNTz‐B**, **IP‐FNTz‐B**.

**Figure 2:**
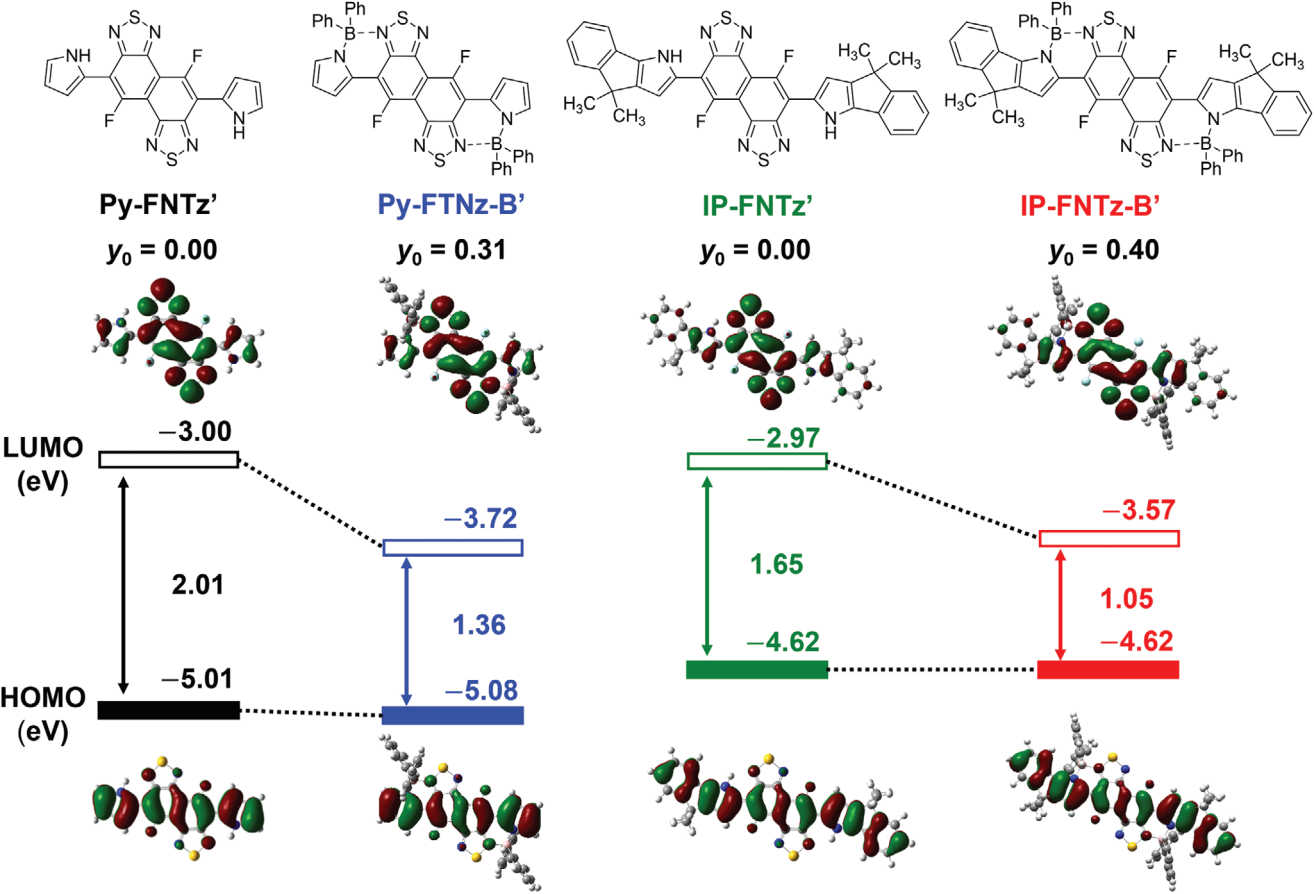
Calculated Kohn‐Sham molecular orbitals, energy diagrams, and *y*_0_ values of **Py‐FNTz’**, **Py‐FNTz‐B’**, **IP‐FNTz’**, and **IP‐FNTz‐B’**.

To clarify the electronic states and the energy levels of **Py‐FNTz‐B** and **IP‐FNTz‐B** as well as the corresponding π‐conjugated framework without B‐N linkages (structures of **Py‐FNT** and **IP‐FNTz**, as shown in **Scheme** **1**), density functional theory (DFT) calculations were conducted at the B3LYP/6‐31g(d,p) level (**Figure** **2**). All the alkyl groups were replaced with either hydrogen atoms or methyl groups to reduce the computational cost. Thus, these were denoted as **Py‐FNTz‐B’**, **IP‐FNTz‐B’**, **Py‐FNTz’**, and **IP‐FNTz’**. As shown in Figure 2, the highest occupied molecular orbitals (HOMOs) of these molecules were distributed across the entire π‐conjugated frameworks, while the lowest unoccupied molecular orbitals (LUMOs) were localized on the FNTz unit due to its strong electron‐accepting characteristics. The HOMO energy levels of **Py‐FNTz‐B’** and **IP‐FNTz‐B’** were almost the same as those of **Py‐FNTz’** and **IP‐FNTz’**. On the other hand, the LUMO energy levels of **Py‐FNTz‐B’** (−3.72 eV) and **IP‐FNTz‐B’** (−3.57 eV) were relatively low‐lying compared with those of **Py‐FNTz’** (−3.00 eV) and **IP‐FNTz’** (−2.97 eV). As a result, **Py‐FNTz‐B’** (1.36 eV) and **IP‐FNTz‐B’** (1.05 eV) showed a narrow HOMO‐LUMO energy gap, compared with those of **Py‐FNTz’** (2.01 eV) and **IP‐FNTz’** (1.68 eV). Focusing on the extension of π‐conjugation showed that the HOMO‐LUMO energy gap of **IP‐FNTz‐B’** was narrower by 0.31 eV than that of **Py‐FNTz‐B’**, which indicated that utilization of the π‐extended D unit is effective for narrowing the HOMO‐LUMO energy gap.

We used UHF/6‐31g(d) to estimate the diradical character (*y*_0_)^[^ ^12^ ^]^ of these D‐A‐D compounds because some NIR absorption dyes show resonance between a closed‐shell aromatic state and an open‐shell diradical singlet ground state.^[^ ^13^ ^]^ In this estimation, the *y*_0_ value of a completely closed‐shell state was calculated as 0, while the open‐shell single diradical state was 1. As shown in Figure 2, the *y*_0_ values of **Py‐FNTz’** and **IP‐FNTz’** were determined to be 0.00. On the other hand, the narrow bandgap molecules of **Py‐FNTz‐B’** and **IP‐FNTz‐B’** showed moderate *y*_0_ values of 0.31 and 0.40, respectively.

### 2.2 Synthesis and Thermal Properties

The processes for the syntheses of **Py‐FNTz‐B** and **IP‐FNTz‐B** appear in Scheme 1. First, we synthesized the pyrrole and indenopyrrole terminal units for these molecules. Pyrrole unit **3** was obtained from 3‐hexylpyrrole (**1**) via protection from a *tert*‐buthoxycarbonyl (Boc) group and a subsequent regioselective borylation. To construct the indenopyrrole unit **10**, 1,4‐dihydroindeno[1,2‐*b*]pyrrole (**7**) was synthesized from 1‐(*p*‐toluenesulfonyl)pyrrole (**5**) through Friedel‐Craft acylation with 2‐iodobenzoyl chloride, intramolecular cyclization by Suzuki‐Miyaura coupling, and a reduction of the carbonyl group using LiAlH_4_. Via protection of the pyrrole N‐H proton of **7** using a Boc group, alkylation, and the following borylation reactions were conducted to provide **10**. Next, Suzuki‐Miyaura coupling between **FNTz‐Br** and **3** in the presence of Pd(dppf)Cl_2_·CH_2_Cl_2_ afforded **Py‐FNTz** in a 67% yield. Note that the deprotection of the Boc groups proceeded smoothly under heated conditions. Finally, intramolecular B‐N linkages were formed by the reaction of **Py‐FNTz** with triphenylborane (BPh_3_) in toluene at 130 °C under a nitrogen atmosphere to provide **Py‐FNTz‐B**. **IP‐FNTz‐B** was also prepared via the same synthetic protocol. Notably, **IP‐FNTz‐B** showed sufficient solubility against common solvents such as toluene, chloroform, and chlorobenzene. All the new compounds were unambiguously identified and characterized via ^1^H and ^13^C NMR spectroscopy as well as by high‐resolution mass spectroscopy (HRMS).

To investigate the thermal stabilities, thermogravimetric analysis (TGA) was conducted for **Py‐FNTz‐B** and **IP‐FNTz‐B** (Figure S1, as shown in the Supporting Information). The TGA curves of **Py‐FNTz‐B** and **IP‐FNTz‐B** showed a 5% weight loss at 250 and 265 °C, respectively, which indicates that these π‐conjugated frameworks possess thermal stability adequate for application to organic semiconducting materials.

### 2.3 X‐ray Analyses

**Figure 3:**
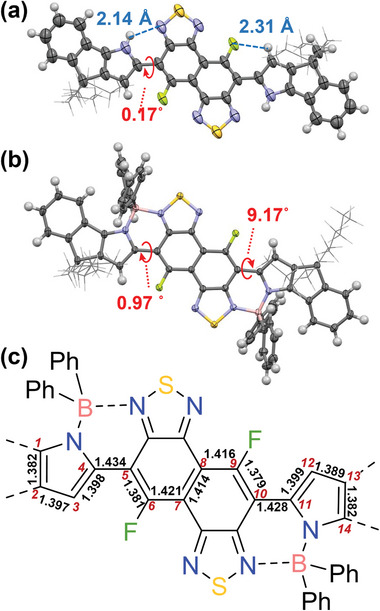
Single‐crystal structures of a) **IP‐FNTz** and b) **IP‐FNTz‐B**. c) Bond length information of **IP‐FNTz‐B**.

To investigate the influence that boron linkages exert on the molecular structures, single‐crystal X‐ray diffraction was conducted for **IP‐FNTz** and **IP‐FNTz‐B**.^[^ ^14^ ^]^ The single crystals of **IP‐FNTz** and **IP‐FNTz‐B** were slowly grown by vapor diffusion with chlorobenzene/methanol. The monomer structures appear in **Figure** **3**a,b and Figure S2 (Supporting Information). **IP‐FNTz** showed a highly planar structure with a dihedral angle of 0.17°, which was due to the intramolecular hydrogen bonding between N‐H‐N (2.14 Å) and C‐H‐F (2.31 Å). **IP‐FNTz‐B** also showed a planar structure with dihedral angles of 0.97° and 9.17°. The B atoms of **IP‐FNTz‐B** were located by a slight displacement of 0.05 and 0.31 Å against the π‐plane of FNTz. Such a displacement has been reported in the structure of quadruply B‐N‐fused dibenzoazaacene.^[^ ^10i^ ^]^ Phenyl groups on the boron atoms and alkyl chains were perpendicularly oriented to the π‐backbone.

We analyzed the influence that the electronic structure of the FNTz unit exerted on the chemical structures. Based on the carbon‐carbon distances of C4‐C5 (1.434 Å), C5‐C6 (1.381 Å), C6‐C7 (1.421 Å), C7‐C8 (1.414 Å), C8‐C9 (1.416 Å), C9‐C10 (1.379 Å), and C10‐C11 (1.428 Å) (Figure 3c), the intramolecular bond length alternative (BLA) of the FNTz unit was estimated using the following equation:

(1) $${\bar{BLA}}_{x-y-z}=\;\frac{\left|l_{1}-l_{2}\right|+\left|l_{2}-l_{3}\right|+\cdot \cdot \cdot \left|l_{n-1}-l_{n}\right|}{\left(n-1\right)}$$

In that equation, the ${\bar{BLA}}_{x-y-z}$ parameter is defined as the average difference in the distance between the continuous C‐C and C = C bonds in the conjugated path, *n* denotes the number of bonds related to BLA, and *l*_n_ denotes the corresponding bond length.^[^ ^15^ ^]^ The ${\bar{BLA}}_{x-y-z}$ parameter of **IP‐FNTz‐B** from C6‐C11 was determined to be 0.038 Å, which is shorter than that of **IP‐FNTz** (0.052 Å). Distinct bond length alternations similar to the polyene structure were observed in both molecules. In particular, the shorter ${\bar{BLA}}_{x-y-z}$ in **IP‐FNTz‐B** implies a more effective delocalization of π‐electrons such as that in the band structure of doped polyacetylene. This is because the diradical characteristics of **IP‐FNTz‐B** were increased, as shown in Figure S3 (Supporting Information), which is in good agreement with the calculated results.

**Figure 4:**
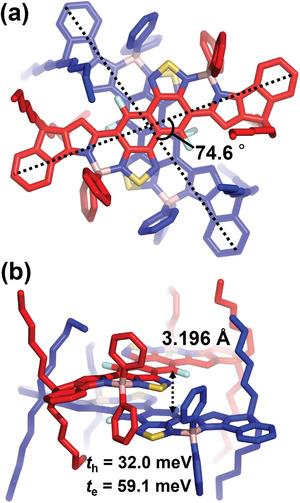
a) Top and b) side views of the packing diagram for **IP‐FNTz‐B**. *t*_h_ and *t*_e_ denote transfer integrals for holes and electrons, respectively.

Focusing on the packing diagram, we found that **IP‐FNTz** formed π‐π stacking structures in the single crystal with the shortest C‐C distance (3.368 Å), as shown in Figure S4 (Supporting Information). By contrast, **IP‐FNTz‐B** formed dimer structures with the central FNTz units placed in a face‐to‐face stacking order with twisted angles of 74.6°, as shown in **Figure** **4**a. In addition, the shortest intermolecular C9‐C9 distance was determined to be 3.196 Å (Figure 4b), which is significantly shorter than the sum of the van der Waals radii of carbon and carbon atoms (C‐C: 3.40 Å). This result indicated the presence of a relatively strong π‐π intermolecular interaction between the π‐planes in the dimer structure. DFT calculations predicted a moderate diradical characteristic for **IP‐FNTz‐B** of 0.40 (Figure 2) and a spin density that is localized mainly on the central NTz unit, as shown in Figure S5 (Supporting Information). Therefore, we assumed that the short π‐π stacked distance of **IP‐FNTz‐B** was caused by the radical‐radical interaction.^[^ ^16^ ^]^ Such a short and strong π‐π stacking of **IP‐FNTz‐B** has an advantage that allows the transport of charge carriers between the molecules. To investigate the influence of the stacking structures on the charge transport characteristics, Amsterdam Density Functional (ADF) calculations were conducted to estimate the transfer integral at the PW91/TZP level. The transfer integrals of the dimer fragment extracted from the crystal structure of **IP‐FNTz** were calculated to be 5.3 meV for the holes (*t*_h_) and 34.5 meV for the electrons (*t*_e_) as shown in Figure S4 (Supporting Information). On the other hand, the corresponding transfer integrals of **IP‐FNTz‐B** were estimated to be 32.0 meV for *t*_h_ and 59.1 meV for *t*_e_, which are larger than the corresponding values for **IP‐FNTz**. This result indicates that the introduction of B‐N linkages contributed to increasing the transfer integrals due to the formation of closed packing structures.

### 2.4 Photophysical Properties

**Figure 5:**
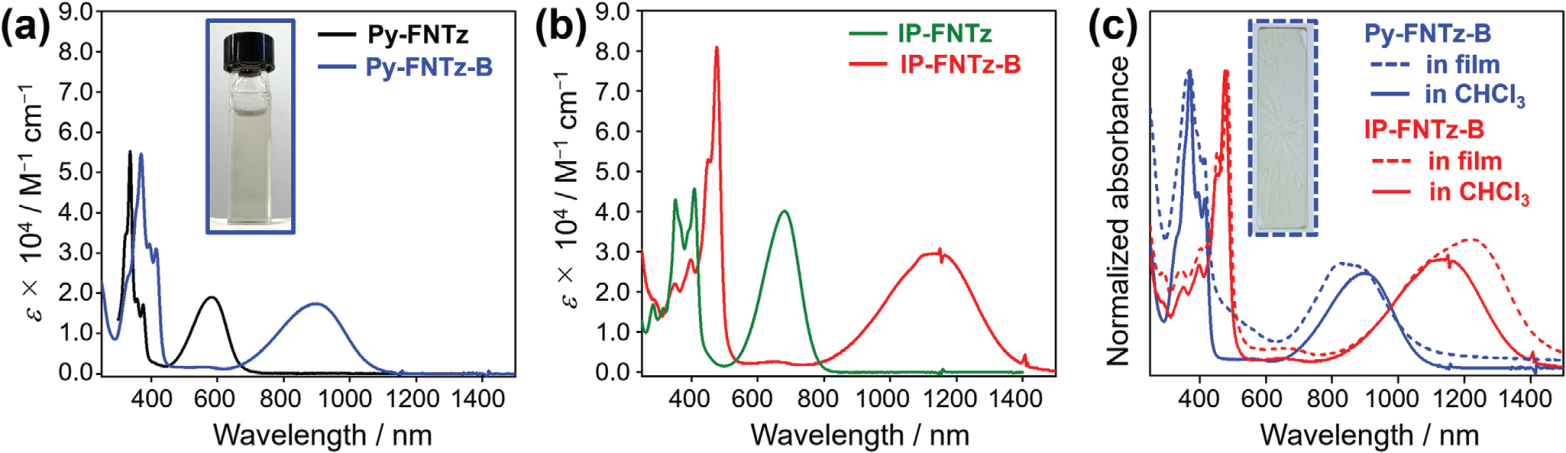
UV–Vis‐NIR absorption spectra of a) **Py‐FNTz** (black) and **Py‐FNTz‐B** (blue), and that of b) **IP‐FNTz** (green) and **IP‐FNTz‐B** (red) in chloroform solutions. The inset picture denotes a solution of **Py‐FNTz‐B**. c) Normalized absorption spectra of **Py‐FNTz‐B** (blue) and **IP‐FNTz‐B** (red) in the solution (solid lines) and in the film state (dotted lines). The Inset picture denotes a thin film of **Py‐FNTz‐B**.

The UV–Vis‐NIR absorption spectra of **Py‐FNTz**, **IP‐FNTz**, **Py‐FNTz‐B**, and **IP‐FNTz‐B** in chloroform solutions appear in **Figure** **5**a,b, and as well as in photophysical data summarized in **Table** **1**. **Py‐FNTz** showed an absorption band with a peak top of 583 nm in the visible region, which resulted in a solution with a blue color. On the other hand, upon the formation of the B‐N linkage, the absorption band that corresponds to the π‐π* transition was significantly red‐shifted toward the NIR region for **Py‐FNTz‐B**, and its maximum absorption wavelength (*λ*_max_) reached 900 nm. Based on the calculated results shown in Figure 2, we considered such a significant red‐shift of **Py‐FNTz‐B** would have derived from the deepened LUMO energy levels. In addition, there were no apparent absorption bands (< 2000 M^−1^ cm^−1^) in the visible region of around 450–650 nm. Eventually, **Py‐FNTz‐B** showed an almost colorless solution. A similar tendency was also observed for **IP‐FNTz** and **IP‐FNTz‐B**; namely, the *λ*_max_ value of **IP‐FNTz‐B** reached 1091 nm, which showed a bathochromic shift by ≈400 nm compared with that of **IP‐FNTz**.

**Table 1:** Summary of physical properties.

Compd.	*λ*_max_ (*ε*) [nm]^a)^	Δ*E*_opt_ [eV]^b)^	*E*_red_ [V]^c)^	*E*_ox_ [V]^c)^	*IP* [eV]^d)^	*EA* [eV]^e)^
**Py‐FNTz**	583 (1.9 × 10^4^)	–	−1.52	0.23	–	–
**Py‐FNTz‐B**	900 (1.7 × 10^4^)	1.17	−0.88	0.36	5.62	3.93
**IP‐FNTz**	681 (4.0 × 10^4^)	–	−1.47	0.18	–	–
**IP‐FNTz‐B**	1134 (3.0 × 10^4^)	0.87	−0.90	0.10	5.27	4.03

^a)^ In chloroform. Values in parentheses are molar extinction coefficients (*e*);

^b)^ Determined from the onset of the absorption spectra in films;

^c)^ Determined by DPV in dichloromethane containing 0.1 m of TBAPF_6_. *E*_red_ and *E*_ox_ denote the first reduction and oxidation potentials, respectively;

^d)^ Estimated using PYSA;

^e)^ Estimated using LEIPS.

We also measured the absorption spectra in neat **Py‐FNTz‐B** and **IP‐FNTz‐B** thin films (Figure 5c). The absorption spectra of **Py‐FNTz‐B** and **IP‐FNTz‐B** appeared in an absorption band with peak tops at 827 and 1134 nm, respectively. **Py‐FNTz‐B** formed colorless films, which maintained the colors of the solution. Since the onset of **IP‐FNTz‐B** reached 1500 nm, this molecule showed significantly narrow optical energy gaps (*ΔE*_opt_) of 0.87 eV in the film state. This result was attributed to the strong intermolecular interactions between the π‐planes due to the concentration of molecules.

### 2.5 Physicochemical Properties

**Figure 6:**
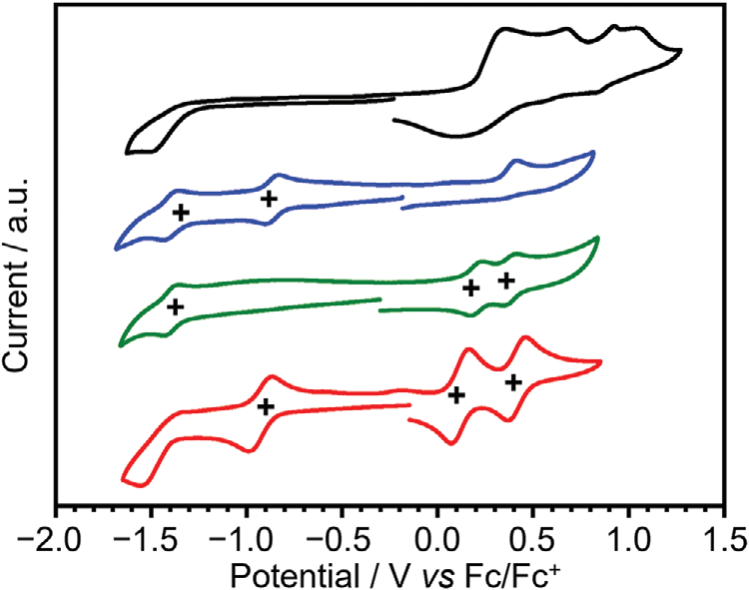
Cyclic voltammograms for **Py‐FNTz** (black), **Py‐FNTz‐B** (blue), **IP‐FNTz** (green), and **IP‐FNTz‐B** (red) in dichloromethane containing 0.1 M TBAPF_6_.

The electrochemical properties of **Py‐FNTz**, **Py‐FNTz‐B**, **IP‐FNTz**, and **IP‐FNTz‐B** were investigated via cyclic voltammetry (CV) and differential pulse voltammetry (DPV) measurements. All the measurements were conducted in a dichloromethane solution containing 0.1 M tetra‐*n*‐butylammonium perfluorophosphate (TBAPF_6_) as a supporting electrolyte, and the oxidation/reduction potentials were referenced against ferrocene/ferrocenium (Fc/Fc^+^) as an internal standard. Oxidation and reduction potentials were determined by the top peak of DPV, as shown in Figure S6 (Supporting Information). Focusing on the anodic scan, **Py‐FNTz** and **Py‐FNTz‐B** show irreversible oxidation waves in **Figure** **6**. This irreversibility indicates that **Py‐FNTz** would undergo polymerization under electrochemical oxidation because a dark‐colored film was formed on the working electrode after repeated CV cycles. On the other hand, **IP‐FNTz** and **IP‐FNTz‐B** showed 2 reversible oxidation waves, which indicates the stable formation of radical cationic and dicationic species. Since the α‐positions of pyrroles are end‐capped by the fused structure with phenyl groups, the decomposition of cationic species was suppressed. In the cathodic scan, **Py‐FNTz** showed 1 irreversible and 2 reversible reduction waves. On the other hand, **IP‐FNTz** and **IP‐FNTz‐B** showed 1 reversible reduction wave. The first reduction potentials, *E*_red_, of both **Py‐FNTz‐B** and **IP‐FNTz‐B** appeared at −0.88 and −0.90 V, respectively, as these were anodically shifted compared with those of **Py‐FNTz** (−1.52 V) and **IP‐FNTz** (−1.47 V) (Table 1). Based on the assumption that Fc/Fc^+^ is below 4.8 eV from the vacuum level, the HOMO/LUMO energy levels were estimated to be −3.28/−5.03 eV for **Py‐FNTz**, −3.92/−5.16 eV for **Py‐FNTz‐B**, −3.33/−4.98 eV for **IP‐FNTz**, and −3.90/−4.90 eV for **IP‐FNTz‐B**. These results indicate that the introduction of boron bridges efficiently lowers the LUMO energy levels, which is consistent with the qualitative trend observed in the DFT calculations.

**Figure 7:**
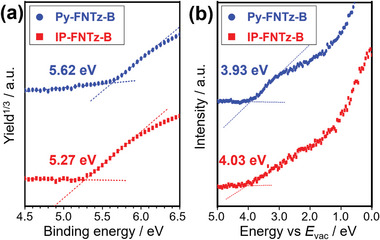
a) PYS and b) LEIPS spectra of **Py‐FNTz‐B** (blue) and **IP‐FNTz‐B** (red) in the films.

To gain information on the energy level regarding ionization potential (IP) and electron affinity (EA) in the films, photoelectron yield spectroscopy (PYS) and low‐energy inverted photoemission spectroscopy (LEIPS) were used. As shown in **Figure** **7**, IP/EA was determined to be 5.62/3.93 eV for **Py‐FNTz‐B** and 5.27/4.03 eV for **IP‐FNTz‐B**. The observed EA values indicated that these molecules have the potential to function as n‐type organic semiconducting materials.

### 2.6 Semiconducting Characteristics

To investigate whether **Py‐FNTz‐B** and **IP‐FNTz‐B** possess intrinsic carrier‐transport characteristics at the nanometer level, time‐resolved microwave conductance (TRMC) measurements were performed for the films prepared on quartz substrates.^[^ ^17^ ^]^ The microwave frequency and its power were ≈9 GHz and ≈3 mW, respectively. A third harmonic generation (355 nm) of a Nd:YAG laser (Continuum Inc., Surelite II, 5−8 ns pulse duration, 10 Hz) was used for the excitation (incident photon density *I*_0_ = 9.1 × 10^15^ photons cm^–2^ pulses^–1^). The photoconductivity (Δ*σ* = *A* ^−1^ Δ*P*_r_ *P*_r_^–1^ where *A* is the sensitivity factor, *P*_r_ is the reflected microwave power, and Δ*P*_r_ is the change in *P*_r_ upon exposure to light) was converted into the product of the quantum yield (*φ*) and sum of the hole mobility (*µ*_h_) and electron mobility (*µ*_e_), Σ*µ* ( = *µ*_h_ + *µ*_e_), using the relationship *φ*Σ*µ* = Δ*σ*(*eI*_0_ *F*_light_)^–1^, where *e* and *F*_Light_ are the electron charge and correction (or filling) factors, respectively.

**Figure 8:**
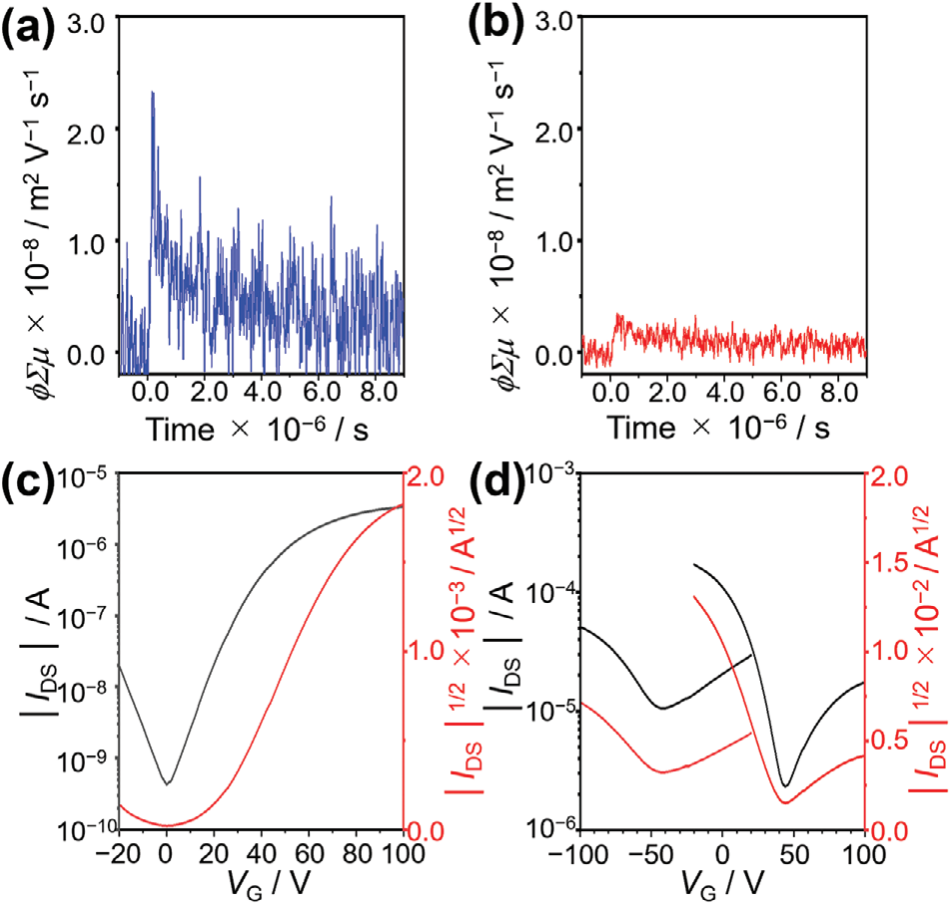
TRMC profiles of a) **Py‐FNTz‐B** and b) **IP‐FNTz‐B**. Transfer characteristics of c) **Py‐FNTz‐B** (*V*_DS_ = 100 V) and d) **IP‐FNTz‐B** (*V*_DS_ = 100 V for n‐channel opperation and *V*_DS_ = −100 V for p‐type operation).

The experiments were performed at room temperature under air. Thin films were prepared via spin‐coating using chloroform solutions of **Py‐FNTz‐B** and **IP‐FNTz‐B**. As shown in **Figure** **8**a,b, transient photoconductivity was observed in both compounds, indicating the possibility of semiconducting characteristics. The value of *φ*Σ*µm*_ax_ for **Py‐FNTz‐B** was determined to be 1.2 × 10^−8^ m^2^ V^−1^ s^−1^, which is higher than that of **IP‐FNTz‐B** (2.5 × 10^−9^ m^2^ V^−1^ s^−1^).

Bottom‐gate, bottom‐contact types of transistor devices were utilized to evaluate the semiconducting characteristics of thin films at the micrometer level, and the active layers were fabricated via spin‐coating onto an octadecyltrichlorosilane (ODTS)‐modified Si/SiO_2_ substrate. The optimized result is listed in Table S1 (Supporting Information). The **Py‐FNTz‐B** film on an ODTS‐modified SiO_2_/Si substrate showed typical n‐type characteristics with a maximum electron mobility (*µ*_e,max_) of 2.4 × 10^−5^ cm^2^ V^−1^ s^−1^, a threshold voltage (*V*_th_) of 20 V, and a current on/off ratio (*I*_on_/*I*_off_) of 10^4^, as shown in Figure 8c and Figure S7 (Supporting Information). On the other hand, the **IP‐FNTz‐B** film showed both p‐ and n‐type characteristics (Figure 8d; Figure S8, Supporting Information). The hole and electron mobilities were determined to be 2.0 × 10^−4^ and 9.5 × 10^−5^ cm^2^ V^−1^ s^−1^, respectively (Table S2, Supporting Information). These mobilities of **Py‐FNTz‐B** and **IP‐FNTz‐B** are relatively higher than those of other reported π‐conjugated derivatives incorporating a BPh_2_ bridge (10^−7^–10^−6^ cm^2^ V^−1^ s^−1^).^[^ ^18^ ^]^

To clarify the differences in the mobilities between **Py‐FNTz‐B** and **IP‐FNTz‐B**, atomic force microscopy (AFM) measurement and X‐ray diffraction were conducted. Based on analysis of the AFM measurement, the **Py‐FNTz‐B** film showed relatively rough morphologies with sub‐micrometer grains (Figure S9a, Supporting Information) while the **IP‐FNTz‐B** film showed grains larger than 1 mm (Figure S10a, Supporting Information). The out‐of‐plane (XRD) characterization of the **Py‐FNTz‐B** film showed several peaks around 2*θ *= 2–5°, which indicated the formation of a polycrystalline film (Figure S9b, Supporting Information). On the other hand, the **IP‐FNTz‐B** film showed a peak at 5.72°, which corresponded to a *d*‐spacing of 15.7 Å (Figure S10b, Supporting Information). These results indicated that **IP‐FNTz‐B** formed a higher orientation in the film state compared with that of **Py‐FNTz‐B**, which resulted in a higher level of carrier mobility.

**Figure 9:**
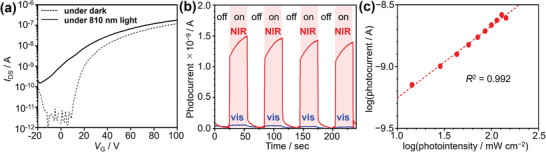
a) Transfer characteristics in the dark (solid line) and under 810 nm light (broken line) at *V*_DS_ = 100 V. b) Time‐dependent switching response profile of the **Py‐FNTz‐B**‐based devices in the dark and under NIR light (red line) at 810 nm or under room LED light (blue line) including visible light. c) Photointensity dependence on photocurrent. (b) and (c): *V*_G_ = 0 V, *V*_DS_ = 100 V.

By taking advantage of the NIR‐selective absorption and n‐type semiconducting characteristics of **Py‐FNTz‐B**, the OPT characteristics were evaluated in the dark and under NIR light illumination of 810 nm at an intensity of 143 mW cm^−2^ using light‐emitting diodes (LEDs). As shown in **Figure** **9**a, the drain‐source current (*I*_DS_) was enhanced up to 10^3^‐fold under irradiation. On the other hand, this device was almost inactive under the illumination of visible light using room‐light LEDs, which indicates the NIR selective detection (Figure 9b). To reveal the current enhancement factor, the dependence of photo intensity on *I*_DS_ was investigated. As shown in Figure 9c and Figure S11 (Supporting Information), the photocurrent, defined as *I*_light_−*I*_dark_, was linearly increased against photo intensity under *V*_G_ = 0 V. This result indicates that the **Py‐FNTz‐B**‐based device functioned as a phototransistor. Figure S12 (Supporting Information) shows the photosensitivity (*P*), photodetectivity (D*), and photoresponsivity (*R*) values as functions of the gate voltage *V*_G_. Based on this result, the maximum photosensitivity *P* was estimated to be 10^3^, shown by the equation *P* = (*I*_light_ − *I*_dark_)/*I*_dark_. The specific detectivity (D*) is given by

(2) $$D^{*}\;=\;\frac{R\sqrt{S}}{I_{N}}$$

where *R* is the photoresponsivity, *S* is the channel area, *I*_N_ is the noise spectral density.^[^ ^19^ ^]^ Since the shot noise of a photodetector is dominated by the dark current (*I*_dark_) in the device, the noise spectral density can be approximately expressed as follows:

(3) $$I_{N}=\;\sqrt{2qI_{dark}}$$

where *q* is the electron charge, *I*_dark_ is the dark current.^[^ ^19^ ^]^ According to Equations (2) and (3), D* value was determined to be 8.9 × 10^9^ Jones. It should be noted that the D* would potentially provide an overestimated value if the observed dark current were higher than the noises.^[^ ^20^ ^]^ Photodetectivity *R* was determined to be 4.2 mA W^−1^, as shown in Figure S12c (Supporting Information). Time‐dependent photoresponses were measured under NIR light at 810 nm with on/off switching at 30 s intervals under 0 V of *V*_G_ and 100 V of *V*_DS_. As shown in Figure 9b, this device showed stable responses with repeated amplifications and suppression of *I*_DS_. These results indicated that **Py‐FNTz‐B** based on the D‐A‐D structure with B‐N linkage has the potential for application as OPTs selective for NIR light.

To measure the charge‐transport characteristics of the **IP‐FNTz‐B** film against a sandwich‐type device with a vertical carrier‐transport direction, we also fabricated space‐charge‐limited current (SCLC) devices that consisted of ITO/ZnO/**IP‐FNTz‐B**/Ca/Al. The *J*–*V* plots appear in Figure S13a (Supporting Information), and the SCLC electron mobilities were determined to be 1.3 × 10^−7^ cm^2^ V^−1^ s^−1^. The active layer in the inset of Figure S13a (Supporting Information) shows almost transparent properties. As shown in Figure S13b (Supporting Information), the transmittance spectra of the active layer on ZnO/ITO showed weak absorption bands at less than 500 nm and at more than 1000 nm and thus showed a high transmittance of more than 80% in the 500–1000 nm region. Although fabricating devices utilizing transparent metal electrodes falls beyond the scope of this study, these results indicate the potential for realizing transparent devices.

### 2.7 Analyses of Electronic Transitions

**Figure 10:**
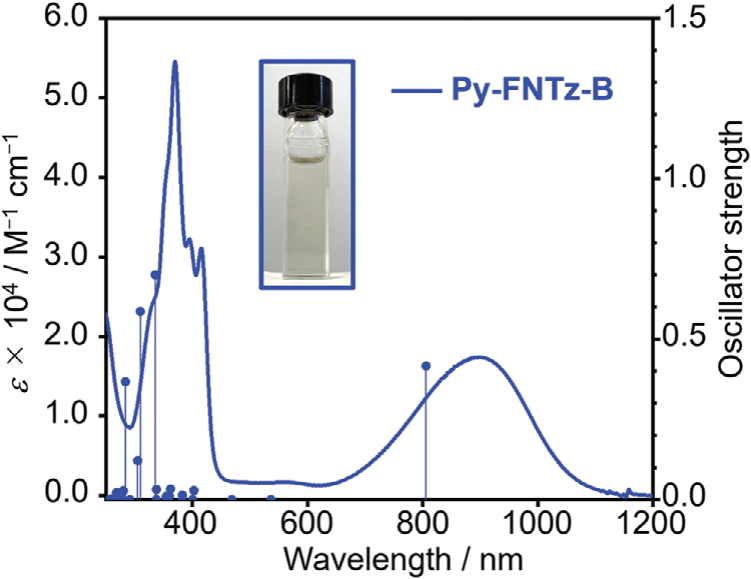
Absorption spectrum (solid line) and oscillator strength (blue circle with dropline) of **Py‐FNTz**. The oscillator strength was estimated at the CAM‐B3LYP/6‐31g(d,p) level.

Both **Py‐FNTz‐B’** and **L2B2** possess the same symmetrical structure with B‐N linkages, which belong to the point group as *C*_2h_. However, there were distinct differences in visible light absorption. To unveil the origin of the significantly different photophysical properties, the details of the electronic transition states were theoretically analyzed using time‐dependent (TD)‐DFT calculations at the CAM‐B3LYP/6‐31g(d,p) level. As summarized in **Table** **2**, the first excited state (S_1_) of **Py‐FNTz‐B’** was the main contribution to the HOMO‐LUMO transition, and the corresponding wavelength was estimated to be 806 nm with an oscillator strength (*f*) of 0.417. The second (S_2_) and third (S_3_) excited states were attributed mainly to the HOMO‐LUMO+1 and HOMO−1‐LUMO transitions, respectively, and these wavelengths were estimated to be 538 and 469 nm, respectively. However, their *f* values were determined to be 0.000. These represent the prevention of transitions and indicate that there is little absorption in the visible region for **Py‐FNTz‐B’**, where the appearance of a colorless state was expected. These simulated transitions of **Py‐FNTz‐B’** well reproduced the absorption spectra, as shown in **Figure** **10**. On the other hand, irrespective of a similar structure such as **Py‐FNTz‐B’**, the **L2B2** molecule showed the allowance of electronic transitions in both the S_1_ and S_3_ states (Table 2). This result indicates that **L2B2** shows absorption bands in both the visible and NIR regions, which also is consistent with the experimental results.

**Table 2:** List of electronic transitions for **Py‐FNTz‐B’** and **L2B2**.

Compd.	ES^a)^	*λ* [nm]	*f*	Major contributions^b)^	Electronic transition
	S_1_	806	0.417	HOMO to LUMO (97%)	*A*_u_ to *B*_g_ : Allowed
**Py‐FNTz‐B’**	S_2_	538	0.000	HOMO to LUMO+1 (68%)	*A*_u_ to *A*_u_ : Forbidden
	S_3_	469	0.000	HOMO−1 to LUMO (67%)	*B*_g_ to *B*_g_ : Forbidden
	S_1_	760	0.242	HOMO to LUMO (95%)	*B*_g_ to *A*_u_ : Allowed
**L2B2**	S_2_	698	0.000	HOMO to LUMO+1 (94%)	*B*_g_ to *B*_g_ : Forbidden
	S_3_	569	0.323	HOMO−1 to LUMO (93%)	*B*_g_ to *A*_u_ : Allowed

^a)^ ES denotes excited state;

^b)^ Values in parenthesis are the contribution of electronic transition.

To determine the reason for such a difference in the electronic transitions between **Py‐FNTz‐B’** and **L2B2**, we focused on the molecular symmetries at each of the energy levels of **Py‐FNTz‐B’** and **L2B2**. Based on rules for the quantum mechanics of selection, the possibility of electronic transition is described in Equation (4).^[^ ^21^ ^]^

(4) $$\int \varphi_{i}\mu \varphi_{f}d\tau$$

In Equation (4), φ_*i*_ and φ_*f*_ denote the wave functions at the initial and final states, respectively, and µ is the electronic transition moment. When the integral value in Equation (4) becomes 0, the electronic transition is forbidden. On the other hand, a value other than 0 allows the transition. This difference can be determined by the change in the orbital symmetry when there is an electronic transition, which is referred to as the Laporte rule.^[^ ^22^ ^]^ According to this rule, parity of the quantum state is essential for transition. For example, orbital symmetries in the initial and final states in a symmetrical orientation are referred to as gerade (as subscript g) (an asymmetrical orientation is referred to as ungerade (as subscript u)), in which case the corresponding electronic transition is forbidden. On the other hand, when the orbital symmetry is inverted from g to u, the electronic transition should be allowed.

**Figure 11:**
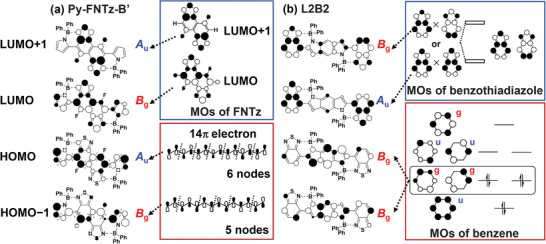
A diagram of the frontier orbital correlations of a) **Py‐FNTz‐B’** and b) **L2B2**.

Since the point group of both **Py‐FNTz‐B’** and **L2B2** was assigned *C*_2h_ symmetry, the symmetry of the molecular skeleton was not related to an electronic transition. Instead, we assumed that it would be important to focus on the orbital symmetries of energy levels in order to establish the possibility of electronic transition. In the case of **L2B2**, the HOMO and HOMO−1 orbitals were localized mainly on the central benzodipyrrole (BDP) unit, as shown in **Figure** **11**b and Figure S14 (Supporting Information). The degeneration of the HOMO energy level in the intrinsic benzene ring was divided into HOMO and HOMO−1 orbitals due to the perturbation that originated from the fused pyrrole rings. Hence, the point groups of both HOMO and HOMO−1 orbitals are considered to be *B*_g_. On the other hand, the LUMO and LUMO+1 orbitals were dominant on the terminal benzothiaziazole units. Therefore, the LUMO orbital is considered to be *A*_u_ due to the in‐phase orientation, while the LUMO+1 orbital is considered to be *B*_g_ due to an antiphase orientation. As a result, not only the S_1_ state of the HOMO‐LUMO transition but also the S_3_ state of the HOMO−1‐LUMO transition was allowed (Table 2), which resulted in a low level of selective absorption.

The HOMO−1 and HOMO of **Py‐FNTz‐B’** were delocalized over the π‐conjugated backbone (Figure 11a; Figure S14a, Supporting Information). Since these orbital arrangements are regarded as the 14−electron system based on a polyene structure, the HOMO−1 and HOMO possess 5 and 6 nodes, respectively (Figure 11a). Hence, the HOMO−1 and HOMO of **Py‐FNTz‐B’** were considered to be *B*_g_ and *A*_u_, respectively. In contrast, the LUMO and LUMO+1 were localized on the central FNTz unit. Hence, based on the orbital symmetry of the FNTz unit, the LUMO and LUMO+1 symmetries were considered to be *B*_g_ and *A*_u_, respectively. As a result, the S_2_ transition of HOMO‐LUMO+1 and the S_3_ transition of HOMO−1‐LUMO are symmetrically forbidden, while the HOMO‐LUMO transition is allowed (Table 2), which led to NIR‐wavelength selective absorption and a colorless behavior.

Based on the tuning of orbital interactions and orbital symmetries, the incorporation of polyene structures enables the design of molecules possessing NIR selective absorption. As summarized in Figure S15 (Supporting Information), this molecular design also allows us to rationalize the transparency of the reported compounds. Therefore, we have considered that this concept is effective for designing NIR selective absorbing dyes with colorless and transparent features. Additionally, it is helpful for interpreting the relationship between absorption properties and molecular structures.

## 3 Conclusion

In summary, to develop colorless NIR‐absorbing dyes for organic electronics, we focused on a molecular design based on controlling electronic transitions. To possess NIR absorption in the NIR region with suppressed π‐π* transitions in the visible region, we designed and synthesized polycyclic D‐A‐D types of π‐conjugated molecules (**Py‐FNTz‐B**, **IP‐FNTz‐B**, and **IP‐FNTz‐B**), which contain pyrrole or indenopyrrole as donor units and FNTz as an acceptor unit. Analyses of single‐crystal X‐ray diffraction for **IP‐FNTz** and **IP‐FNTz‐B** were conducted, and their chemical structures revealed distinct bond length alternations similar to the polyene structure. Moreover, the introduction of a B‐N linkage reduced the bond length alternation, which indicated effective delocalization of the π‐electrons. The pyrrole end‐capped **Py‐FNTz‐B** showed an absorption band in the NIR region at 888 nm without distinct absorption in the visible region, which led to colorless and transparent characteristics in the film state. The indenopyrrole end‐capped **IP‐FNTz‐B** in films showed a narrow *ΔE*_opt_ of 0.87 eV, which is narrower by 0.30 eV than that of **Py‐FNTz‐B**. Transient photoconductivity in the TRMC measurements was observed in both compounds, indicating the possibility of semiconducting characteristics. Eventually, the OFET devices of **Py‐FNTz‐B** showed typical n‐type characteristics and photodetection behaviors under NIR light irradiation at 810 nm. The diode device based on **IP‐FNTz‐B** showed an almost transparent film possessing electron transport characteristics. TD‐DFT calculations showed that **Py‐FNTz‐B** has one allowed electronic transition in the NIR region and 2 forbidden electronic transitions in the visible region. From the analysis of orbital correlations, the orbital symmetries of **Py‐FNTz‐B** from HOMO−1 to LUMO+1 showed an alternating inverted configuration due to the contribution of the polyene‐like structure and the presence of a strong FNTz unit, which led to NIR wavelength‐selective absorption. This study demonstrates that orbital symmetries of the electronic structures in D‐A‐D systems are an important guideline for the molecular design of colorless NIR absorbing dyes, which could contribute to the development of colorless and transparent organic electronics with NIR‐conducive characteristics.

## Conflict of Interest

The authors declare no conflicts of interest.

## Supporting information

Supporting Information

## Data Availability

The data that support the findings of this study are available in the supplementary material of this article.
