# Supplementary material for: Colorless Near‐Infrared Absorbing Dyes Based on B‐N Fused Donor‐Acceptor‐Donor π‐Conjugated Molecules for Organic Phototransistors
Source: Adv Sci (Weinh). 2024 Jun 14;11(31):2405656. doi: 10.1002/advs.202405656 (PMC11336916; doi:10.1002/advs.202405656)
Supplement: Supplementary file 1 — Supporting Information [file ADVS-11-2405656-s001.docx]

Supporting Information

Colorless Near-Infrared Absorbing Dyes Based on B-N Fused Donor-Acceptor-Donor π-Conjugated Molecules for Organic Phototransistors

Soichi Yokoyama*, Sakura Utsunomiya, Takuji Seo, Akinori Saeki, and Yutaka Ie*

**Experimental procedures**

**General Information**

Column chromatography was performed on silica gel (KANTO Chemical silica gel 60N with 40–50 μm mesh for normal phase). Preparative gel permeation chromatography (GPC) was performed on Bio-Beads S-X3 with 40-80 μm bead size. Recycling preparative GPC was performed on a JAPAN Analytical Industry LaboACE LC-5060 equipped with JAI-GEL 1HR/2HR. ^1^H and ^13^C NMR spectra were recorded on a JEOL JNM-ECS600 spectrometer in CDCl_3_, CD_2_Cl_2_ or tetrachloroethane-*d*_2_. Tetramethylsilane (TMS) or solvent peaks were used as an internal standard. Data are reported as follows: chemical shift in ppm (δ), multiplicity (s = singlet, d = doublet, t = triplet, dd = double doublet, m = multiplet), coupling constant (*J*), and integration. It should be noted that several ^13^C NMR peaks were overlapped due to the molecular symmetries of the materials. High-resolution mass spectra (HRMS) were obtained by atmospheric pressure chemical ionization (APCI) methods using a Thermo scientific LTQ Orbitrap XL. All chemicals and reagents were purchased from commercial sources (FUJIFILM Wako Chemicals, TCI, Kanto Chemical and Sigma Aldrich). The 3-hexylpyrrole (**1)** and 1-tosylpyrrole (**4**) were synthesized according to previously established processes found in the literature.^[S1]-[S2]^ UV-vis-NIR spectra were recorded on a Shimadzu UV-3600 spectrophotometer. Thermogravimetric (TGA) analyses were performed under nitrogen at a heating rate of 10 °C min^–1^ with a Shimadzu TGA-50. Cyclic voltammetry (CV) and differential pulse voltammetry (DPV) were carried out on a BAS CV-620C voltammetric analyzer using a platinum disk as the working electrode, platinum wire as the counter electrode, and Ag/AgNO_3_ as the reference electrode, with a supporting electrolyte of 0.1 m tetrabutylammonium hexafluorophosphate (TBAPF_6_) solution in dichloromethane. Electrochemical potentials were referenced to an Fc/Fc^+^ as an internal standard. The surface morphologies of the deposited organic films were observed by atomic force microscopy (AFM) (Shimadzu, SPM9600). The film crystallinity was evaluated by X-ray diffractometer (Rigaku, SmartLab). X-ray diffraction patterns were obtained using Bragg-Brentano geometry with CuK*α* radiation as an X-ray source with an acceleration voltage of 45 kV and a beam current of 200 mA. The scanning mode was set to 2*θ χ/ϕ* scans between 2º–30º with scanning steps of 0.1º, and the incidence angle was fixed at 0.20°. Photoelectron yield spectroscopy (PYS) was performed using a Bunkoukeiki BIP-KV202GD. Low-energy inverse photoemission spectroscopy (LEIPS) was performed using the Ulvac-Phi, Inc. LEIPS system (734170). The films of these measurements were fabricated on ITO substrates by the spin-coating of a solution of **Py-FNTz-B** and **IP-FNTz-B** in CHCl_3_.

**Synthesis Procedures and Characterization**

*Synthesis of* ***2***: To a solution of compound **1**^[S1]^ (0.435 g, 2.88 mmol) in CH_3_CN (15 mL) was added a solution of di-*tert*-butyl dicarbonate (Boc_2_O) (0.754 g, 3.45 mmol) in CH_3_CN (15 mL) at room temperature. After addition of 4-dimethylaminopyridine (DMAP) (35 mg, 0.29 mmol), the reaction mixture was stirred overnight at room temperature. After the addition of water, the mixture was extracted with CHCl_3_. The organic phase was dried over Na_2_SO_4_, filtered, and evaporated in vacuo. The residue was purified by column chromatography on silica gel (hexane:CHCl_3_ = 3:1 as an eluent) to afford **2** as a pale yellow oil. (0.590 g, 2.35 mmol, 81%); ^1^H NMR (400 MHz, CDCl_3_, *δ*) 7.15 (brs, 1H), 6.97 (brs, 1H), 6.08 (dd, *J* = 2.8, 1.2 Hz, 1H), 2.40 (t, *J* = 7.8 Hz, 2H), 1.59 (s, 9H), 1.54-1.50 (m, 2H), 1.37-1.27 (m, 6H), 0.88 (t, *J* = 6.9 Hz, 3H); HRMS (APCI) *m/z*: [M−Boc+2H]^+^: calcd. for C_10_H_18_N^+^, 152.1434; found, 152.1432.

*Synthesis of* ***3***: 1.0 m of LDA (7.0 mL, 7.0 mmol) was slowly added to a solution of compound **2** (249 mg, 0.990 mmol) in THF (20 mL). After stirring for 30 min at −78 °C, 2-isopropoxy-4,4,5,5-tetramethyl-1,3,2-dioxaborolane (*i*-PrO-Bpin) (1.6 mL, 8.0 mmol) was slowly added to the reaction mixture. After stirring for 30 min, the reaction mixture was warmed to 0 ° C, and further stirring was conducted for 30 min. The reaction temperature was warmed to room temperature, and then reaction mixture was stirred overnight. After quenching with water, the reaction mixture was extracted with CHCl_3_. The organic phase was dried over Na_2_SO_4_, filtered, and evaporated in vacuo. The residue was purified by column chromatography on silica gel (hexane:AcOEt = 8:1 as an eluent). The further purification was conducted by GPC recycling using CHCl_3_ as an eluent to afford **3** as a yellow oil. (155 mg, 0.411 mmol, 42%); ^1^H NMR (400 MHz, CDCl_3_, *δ*) 7.07-7.07 (m, 1H), 6.50 (d, *J* = 1.6 Hz, 1H), 2.36 (t, *J* = 7.2 Hz, 2H), 1.58 (s, 9H), 1.56-1.50 (m, 2H), 1.34 (s, 12H), 1.32-1.27 (m, 6H), 0.88 (t, *J* = 7.2 Hz, 3H); ^13^C NMR (101 MHz, CD_2_Cl_2_, *δ*) 150.1, 128.2, 124.3, 121.8, 83.9, 83.6, 32.2, 30.8, 29.4, 28.1, 26.8, 24.9, 23.1, 14.3; HRMS (APCI) *m/z*: [M−Boc]^−^: calcd. for C_16_H_27_BNO_2_, 276.2140; found, 276.2146.

*Synthesis of* ***5***: Aluminum chloride (5.42 g, 40.7 mmol) was added at 0 °C to a solution of 2-iodobenzoyl chloride (10.0 g, 37.5 mmol) in 1,2-dichloroethane (45 mL) under nitrogen. After stirring for 10 min, 1-tosylpyrrole (**4**)^[S2]^ (7.01 g, 31.7 mmol) in 1,2-dichloroethane (70 mL) was added slowly. After stirring overnight at room temperature, the reaction mixture was poured into cold water. Then, the mixture was extracted with dichloromethane. The combined organic phase was dried over MgSO_4_. After evaporation of the solvent, the residue was purified by column chromatography on silica gel (dichloromethane as an eluent). Further purification was conducted by reprecipitation of dichloromethane/hexane to afford **5** as a white solid. (12.9 g, 28.6 mmol, 90%); ^1^H NMR (400 MHz, CDCl_3_, *δ*) 7.92 (dd, *J* = 7.8, 0.9 Hz, 1H), 7.79-7.77 (m, 2H), 7.46-7.42 (m, 2H), 7.35-7.32 (m, 3H), 7.20-7.16 (m, 2H), 6.76 (dd, *J* = 3.6, 1.2 Hz, 1H), 2.43 (s, 3H); ^13^C NMR (101 MHz, CDCl_3_, *δ*) 191.3, 146.3, 144.4, 140.2, 135.0, 131.5, 130.6, 128.4, 128.0, 127.8, 127.7, 127.5, 122.1, 113.4, 92.2, 21.9; HRMS (APCI) *m*/z: [M+H]^+^: calcd. for C_18_H_15_INO_3_S, 451.9812; found, 451.9810.

*Synthesis of* ***6***: *N,N*-Dimethylacetamide (AcNMe_2_) (120 mL) was added to a round-bottom flask containing compound **5** (12.3 g, 27.3 mmol), palladium(II) acetate (0.604 g, 2.69 mmol), and potassium carbonate (7.43 g, 53.8 mmol) under argon atmosphere. After stirring overnight at 140 °C, AcNMe_2_ was evaporated *in vacuo*. Then, 1 m HCl aqueous solution was added to the resulting residue, and the product was extracted with dichlomethane. The organic layer was dried over MgSO_4_, filtered, and evaporated. The product was purified by column chromatography on silica gel (dichloromethane as an eluent) and then column chromatography on GPC gel (THF as an eluent). Further purification was conducted by reprecipitation from dichloromethane/hexane to afford **6** as a red solid. (3.37 g, 19.9 mmol, 73%); ^1^H NMR (400 MHz, DMSO-*d*_6_, *δ*) 11.87 (brs, 1H), 7.28 (t, *J* = 7.6 Hz, 1H), 7.21 (d, *J* = 7.2 Hz, 1H), 7.08 (d, *J* = 7.6 Hz, 1H), 7.05 (d, *J* = 7.2 Hz, 1H), 6.83 (d, *J* = 2.8 Hz, 1H), 6.13 (d, *J* = 2.4 Hz, 1H); ^13^C NMR (101 MHz, DMSO-*d*_6_, *δ*) 185.9, 150.8, 139.4, 136.0, 133.0, 127.5, 125.3, 122.7, 122.6, 117.1, 104.8; HRMS (APCI) *m*/z: [M+H]^+^: calcd. for C_11_H_8_NO,170.0601; found, 170.0601.

*Synthesis of* ***7***: A solution of compound **6** (1.5 g, 8.9 mmol) in THF (90 mL) was added at 0 °C to a solution of LiAlH_4_ (3.7 g, 98 mmol) in THF (50 mL). After refluxing overnight, the reaction mixture was quenched via a saturated Na_2_SO_4_ aqueous solution, and then dried over Na_2_SO_4_. The reaction mixture was filtered and washed with THF, CHCl_3_, and ethyl acetate. After evaporation of the solvent, the residue was purified by column chromatography on silica gel (hexane:CHCl_3_ = 1:3 as an eluent) to afford **7** as a white solid. (1.2 g, 7.7 mmol, 87%); ^1^H NMR (400 MHz, CDCl_3_, *δ*) 8.34 (brs, 1H), 7.43 (d, *J* = 7.2 Hz, 1H), 7.31 (d, *J* = 8.0 Hz, 1H), 7.25 (t, *J* = 7.2 Hz, 1H), 7.08 (td, *J* = 7.2, 1.6 Hz, 1H), 6.88 (d, *J* = 2.4 Hz, 1H), 6.30 (t, *J* = 2.4 Hz, 1H), 3.48 (s, 2H); ^13^C NMR (101 MHz, CDCl_3_, *δ*) 147.3, 137.7, 135.5, 129.2, 126.5, 125.5, 123.3, 121.3, 116.0, 105.1, 30.9; HRMS (APCI) *m*/z: [M+H]^+^: calcd. for C_11_H_10_N, 156.0808; found, 156.0806.

*Synthesis of* ***8***: A solution of Boc_2_O (2.00 g, 9.16 mmol) in CH_3_CN (5 mL) and 4-dimethylaminopyridine (94 mg, 0.77 mmol) was added to a solution of compound **7** (1.19 g, 7.67 mmol) in CH_3_CN (15 mL). After stirring overnight at room temperature, the reaction solution was evaporated. The resulting mixture was purified by column chromatography on silica gel (hexane:CHCl_3_ = 3:1 as an eluent) to afford **8** as a pale yellow oil. (1.81 g, 7.09 mmol, 92%); ^1^H NMR (400 MHz, CDCl_3_, *δ*) 8.20 (d, *J* = 7.6 Hz, 1H), 7.42 (d, *J* = 7.6 Hz, 1H), 7.30 (t, *J* = 7.6 Hz, 1H), 7.26 (d, *J* = 3.2 Hz, 1H), 7.13 (td, *J* = 7.6, 1.2 Hz, 1H), 6.29 (d, *J* = 3.2 Hz, 1H), 3.47 (s, 2H), 1.66 (s, 9H); ^13^C NMR (101 MHz, CDCl_3_, *δ*) 149.2, 147.0, 138.6, 135.5, 133.4, 126.7, 124.9, 124.4, 124.1, 120.8, 108.0, 84.0, 30.9, 28.3; HRMS (APCI) *m*/z: [M+H]^+^: calcd. for C_16_H_18_NO_2_, 256.1333; found, 256.1331.

*Synthesis of* ***9***: A solution of 1-iodehexane (2.8 mL, 19 mmol) in THF (30 mL) was added to a solution of compound **8** (800 mg, 3.13 mmol) in THF (30 mL). After the reaction mixture was cooled to 0 °C, potassium *tert*-butoxide (754 mg, 6.72 mmol) was added at 0 °C. After stirring at 0 °C for 2 hours, the reaction mixture was stirred at room temperature for 2 hours. The reaction mixture was quenched using saturated ammonium chloride aqueous solution, and then extracted with dichloromethane. The organic layer was dried over Na_2_SO_4_, filtered, and then the solvent was removed by evaporation. The residue was purified by column chromatography on silica gel (hexane and hexane:dichloromethane = 3:1 as an eluent) to afford **9** as a pale yellow oil. (873 mg, 2.06 mmol, 66%); ^1^H NMR (400 MHz, CDCl_3_, *δ*) 8.13-8.10 (m, 1H), 7.25-7.23 (m, 3H), 7.19-7.10 (m, 1H), 6.19-6.17 (m, 1H), 1.94-1.87 (m, 2H), 1.77-1.71 (m, 2H), 1.67 (s, 9H), 1.16-1.12 (m, 12H), 0.94-0.78 (m, 10H); ^13^C NMR (101 MHz, CDCl_3_, *δ*) 155.1, 149.3, 141.0, 136.8, 135.1, 126.6, 124.4, 124.2, 122.2, 120.6, 107.3, 83.8, 50.3, 38.9, 31.8, 30.0, 28.3, 24.5, 22.8, 14.2; HRMS (APCI) *m*/z: [M＋H]^+^: calcd. for C_28_H_42_NO_2_, 424.3211; found, 424.3205.

*Synthesis of* ***10***: To a solution of compound **9** (0.280 g, 0.661 mmol) in THF (10 mL) was added 1.0 m of LDA (2.0 mL, 2.0 mmol) in THF/hexanes at −78 °C. After stirring for 30 min at −78 °C, *i*-PrO-Bpin (0.50 mL, 2.47 mmol) was added to the reaction mixture. After stirring or 30 min. −78 °C, the reaction mixture was heated to 0 °C and stirred for 30 min. at 0 °C, and then at room temperature for 60 min. After quenching the reaction by the addition of water, the mixture was extracted with CHCl_3_ and the organic layer was washed with water, and then dried over Na_2_SO_4_. After evaporation of the solvent, the residue was purified by column chromatography on silica gel (hexane:CHCl_3_ = 2:1) to afford **10** as a pale yellow oil. (0.323 g, 0.588 mmol, 89%); ^1^H NMR (600 MHz, CDCl_3_, *δ*) (ppm) 7.98 (d, *J* = 5.2 Hz, 1H), 7.23-7.20 (m, 2H), 7.14-7.12 (m, 1H), 6.66 (s, 1H), 1.87 (td, *J* = 8.4, 2.8 Hz, 2H), 1.71-1.66 (m, 11H), 1.36 (s, 12H), 1.17-1.07 (m, 12H), 0.96-0.93 (m, 2H), 0.81-0.76 (m, 8H); ^13^C NMR (151 MHz, CDCl_3_, *δ*) 156.3, 150.8, 141.5, 141.0, 134.8, 126.4, 124.9, 122.5, 120.7, 118.8, 84.8, 83.7, 49.7, 38.9, 31.8, 30.0, 28.2, 25.0, 24.5, 22.8, 14.2; HRMS (ACPI) *m/z*: [M−Boc+2H]^+^: calcd. for C_29_H_45_BNO_2_, 450.3538; found, 450.3537.

*Synthesis of* ***Py-FNTz***: To a solution of **FNTz-Br** (60.8 mg,0.139 mmol), compound **3** (157 mg, 0.416 mmol), K_2_CO_3_ (58.1 mg, 0.420 mmol) and Pd(dppf)Cl_2_·CH_2_Cl_2_ (5.8 mg, 7.1 μmol) in dioxane (3 mL) and water (1 mL) was stirred overnight at 100 °C. The reaction mixture was cooled to room temperature and extracted with CHCl_3_. The organic layer was dried over MgSO_4_, filtered, and evaporated in vacuo. The resulting residue was passed though column chromatography on silica (CHCl_3_/hexane = 3/1 as an eluent). The protected Boc groups were removed by heating at 185 °C for 6 hours under 2 Torr in Kugelrohr. Then, the product was purified with column chromatogdraphy on silica gel (CHCl_3_/hexane = 1/1 as an eluent) to afford **Py-FNTz** as a dark violet solid. (42.0 mg, 72.6 mmol, 52%); ^1^H NMR (400 MHz, dichloromethane-*d*_2_, *δ*) 11.12 (brs, 2H), 7.23 (s, 2H), 7.97 (s, 2H), 2.60 (t, *J* = 7.2 Hz, 4H), 2.60 (quint, *J* = 7.4 Hz, 4H), 1.44-1.34 (m, 12H), 0.91 (t, *J* = 6.8 Hz, 6H); ^13^C NMR (151 MHz, tetrachloroethane-*d*_2_, *δ*) 154.6, 152.8, 152.0, 146.6, 127.0, 122.6, 118.8, 114.8, 114.8, 114.7, 113.7, 113.6, 113.5, 109.8, 109.7, 31.7, 30.8, 29.2, 26.9, 22.6, 14.1; HRMS (ACPI) *m/z*: [M+H]^+^: calcd. for C_30_H_33_F_2_N_6_S_2_, 579.2171; found, 579.2171.

*Synthesis of* ***Py-FNTz-B***: Triphenylboron (242 mg, 1.00 mmol) was added to a solution **Py-FNTz** (30.1 mg, 52.0 μmol) in toluene (3 mL), the mixture was stirred at 130 °C for 3 days under argon atmosphere in a screw tube. The reaction mixture was cooled to room temperature. The product was purified with column chromatography on silica gel (CHCl_3_ as an eluent). Further purification was conducted by reprecipitation form dichloromethane/hexane to afford **Py-FNTz-B** as a gray solid. (42.0 mg, 46.3 μmol, 89%); ^1^H NMR (600 MHz, tetrachloroethane-*d*_2_, *δ*) 7.27-7.23 (m, 20H), 7.15 (s, 2H), 6.78 (s, 2H), 2.50 (t, *J* = 5.2 Hz, 4H), 1.62 (quint, *J* = 5.2 Hz, 4H), 1.37-1.30 (m, 12H), 0.88 (t, *J* = 4.0 Hz, 6H); ^13^C NMR (151 MHz, tetrachloroethane-*d*_2_, *δ*)153.5, 151.8, 148.3, 148.0, 147.9, 146.1, 132.8, 129.8, 129.6, 127.8, 127.7, 127.3, 122.5, 115.2, 113.2, 113.2, 113.1, 113.1, 109.5, 109.4, 31.6, 30.8, 29.0, 27.0, 22.6, 14.1; HRMS (ACPI) *m/z*: [M+H]^+^: calcd. for C_54_H_51_B_2_F_2_N_6_S_2_, 907.3766; found, 907.3766.

*Synthesis of* ***IP-FNTz***: A solution of **FNTz-Br** (63.0 mg, 0.143 mmol), compound **10** (234 mg, 0.426 mmol), and Pd(dppf)_2_Cl_2_·CH_2_Cl_2_ (5.90 mg, 7.3 μmol) in dioxane (3 mL) and water (1 mL) was stirred overnight at 100 ° C. The reaction mixture was cooled to room temperature and extracted with CHCl_3_. The organic layer was dried over MgSO_4_, filtered, and evaporated in vacuo. The resulting residue was passed though column chromatography on silica (CHCl_3_ as an eluent). The protected Boc groups was removed by heating at 180-185 °C for 2.5 hours under 2 Torr in Kugelrohr. Then, the product was purified with column chromatography on silica gel (CHCl_3_/hexane = 3/1 to 1/2 as an eluent) to afford **IP-FNTz** as a green solid. (87.8 mg, 0.0951 mmol, 66%); ^1^H NMR (600 MHz, dichloromethane-*d*_2_, *δ*) 11.48 (s, 2H), 7.46 (d, *J* = 7.2 Hz, 2H), 7.37 (d, *J* = 6.6 Hz, 2H), 7.29-7.27 (m, 4H), 7.21 (t, *J* = 7.2 Hz, 2H), 2.10 (td, *J* = 13.2, 4.2 Hz, 4H), 1.85 (td, *J* = 13.2, 4.2 Hz, 4H), 1.24-1.16 (m, 28H), 1.0 0-0.96 (m, 4 H), 0.81 (t, *J* = 7.2 Hz, 12H); ^13^C NMR (151 MHz, dichloromethane-*d*_2_, *δ*) 156.7, 154.8, 153.0, 152.3, 152.2, 147.0, 140.2, 139.8, 134.0, 127.3, 127.2, 125.4, 123.4, 117.9, 113.2, 111.1, 111.0, 110.2, 110.1, 51.5, 39.5, 32.3, 30.4, 25.4, 23.2, 14.3; HRMS (ACPI) *m/z*: [M+H]^+^ calcd. for C_56_H_65_F_2_N_6_S_2_, 923.4675; found, 923.4677.

*Synthesis of* ***IP-FNTz-B***: Triphenylboron (202 mg, 0.834 mmol) was added to a solution **IP-FNTz** (40.2 mg, 43.5 μmol) in toluene (3 mL), the mixture was stirred at 130 °C for 2 days under argon atmosphere in a screw tube. The reaction mixture was cooled to room temperature. The product was purified with column chromatography on silica gel (CHCl_3_ as an eluent). Further purification was conducted by reprecipitation from dichloromethane/hexane to afford **IP-FNTz-B** as a black solid. (55.6 mg, 42.4 μmol, 97%); ^1^H NMR (600 MHz, dichloromethane-*d*_2_, *δ*) 7.52 (brs, 8H), 7.28-7.21 (m, 16H), 6.97 (t, *J* = 7.2 Hz, 2H), 6.71 (t, *J* = 7.2 Hz, 2H), 7.16 (d, *J* = 7.8 Hz, 2H), 2.03 (td, *J* =13.2 , 4.2 Hz, 4H), 1.84 (td, *J* =12.6 , 4.2 Hz, 4H), 1.24-1.06 (m, 28 H), 0.91-0.88 (m, 4H), 0.81 (t, *J* = 7.2 Hz); ^13^C NMR (151 MHz, CDCl_3_, *δ*) 156.1, 155.8, 152.5, 150.7, 150.7, 149.3, 146.8, 146.7, 146.7, 146.6, 146.6, 146.5, 146.5, 146.4, 146.4, 146.2, 146.1, 146.1, 146.1, 146.1, 146.0, 145.7, 143.4, 135.0, 133.1, 128.2, 127.6, 127.6, 126.3, 125.1, 122.2, 121.7, 111.1, 111.1, 111.0, 110.5, 110.5, 110.4, 110.4, 110.3, 108.6, 108.5, 49.7, 39.7, 32.0, 30.0, 24.7, 22.8, 14.1; HRMS (ESI) *m/z*: [M]^+^: calcd. for C_80_H_82_B_2_F_2_N_6_S_2_, 1250.6197, found, 1250.6215.

**OFET Device Fabrication and Evaluation**

The field-effect electron mobility was measured using bottom-gate bottom-contact OFET devices. The p-doped silicon substrate functions as the gate electrode. A thermally grown silicon oxide (SiO_2_) dielectric layer on the gate substrate is 300 nm in thickness with a capacitance of 10.0 nF cm^–2^. Interdigital source and drain electrodes were constructed with gold (30 nm) that were formed on the SiO_2_ layer. The channel width (*W*) and channel length (*L*) were 38 mm and 5 μm, respectively. The silicon oxide surface was first washed with toluene, acetone and 2-propanol. It was then activated by ozone treatment and pretreated with octadecyltrichlorosilane (ODTS). The semiconducting layer was fabricated by spin coating 0.25, 0.5 or 1.0 wt% CHCl_3_ solution at 1500 rpm for 1 min or by the spin-coating method onto the substrate in glove box, followed by annealing for 60 min at various temperatures under a vacuum condition (10^–3^ Pa). The characteristics of the OFETs were measured at room temperature under a pressure of 10^–3^ Pa by using a KEITHLEY 4200 semiconductor parameter analyzer. The μ_h_ and μ_e_ was calculated in the saturated region at the *V*_DS_ of 100 V by the following equation:

$$I_{\mathrm{DS}}= \frac{W}{2L}C_{i}\mu(V_{G} - V_{th})^{2}$$

NIR photoirradiation at 810 nm was conducted using a light source (CL-1501, ASAHI Spectra) equipped with LED head (CL-H1-810-9-1). Photosensitivity (*P*) is estimated by the equation *P* = (*I*_light_ − *I*_dark_)/*I*_dark_. The specific detectivity (*D**) is calculated by the equation *D** = *RS*^1/2^/(2*qI*_dark_)^1/2^, where *R* is the photoresponsivity, *S* is the channel area (*LW*), and *q* is the electron charge. In this equation, photoresponsivity *R* is defined as *R* = *J*/*P*_d_. Here, *J* is current density, which can be determined by (*I*_light_−*I*_dark_)/(*WT*), and *P*_d_ is the power of the incident light. *T* denotes the film thickness.

**SCLC Measurements**

Electron-only devices were prepared with a structure of ITO/ITO/ZnO/active layer/Ca/Ag.^[S3]-[S5]^ ITO-coated glass substrates were first cleaned by ultrasonication in acetone, water, and then 2-propanol for 15 min, respectively. After ITO-coated glass substrates were exposed by ozone for 1 h, ZnO layer was spin-coated using the solution of zinc acetate dihydrate (99.9%, 200 mg), ethtanolamine (99%, 55 μL), and 2-methoxyethanol (99.8%, 2 mL) at 3000 rpm, and then baked at 200 °C for 30 min in air. Subsequently, the active layer of **IP-FNTz-B** was formed by spin-coating (0.5 wt% solution in CHCl_3_, 1000 rpm, 1 min) onto an ITO/ZnO electrode in a glove box. Ca and Al electrodes were evaporated on the top of active layer through a shadow mask to define the active area of the devices (0.09 cm^2^) under a vacuum of 10^–5^ Pa to a thickness of 30, 60 nm determined by a quartz crystal monitor. The current density–voltage characteristics of photovoltaic devices were measured by using a KEITHLEY 2400 source meter. The carrier mobilities of these devices were calculated by the following equation:

$J= \frac{9}{8}\varepsilon\varepsilon_{0}\mu\frac{V^{3}}{d^{3}}$

where *ε*, *ε*_0_, μ, and *d* are the dielectric constant of the active layer, the permittivity of free space, the carrier mobility, and the measured thickness of active layer (64.8 nm), respectively. We used the values of *ε* = 3, *ε*_0_ = 8.8 × 10^–12^.

**Supplemental Data**


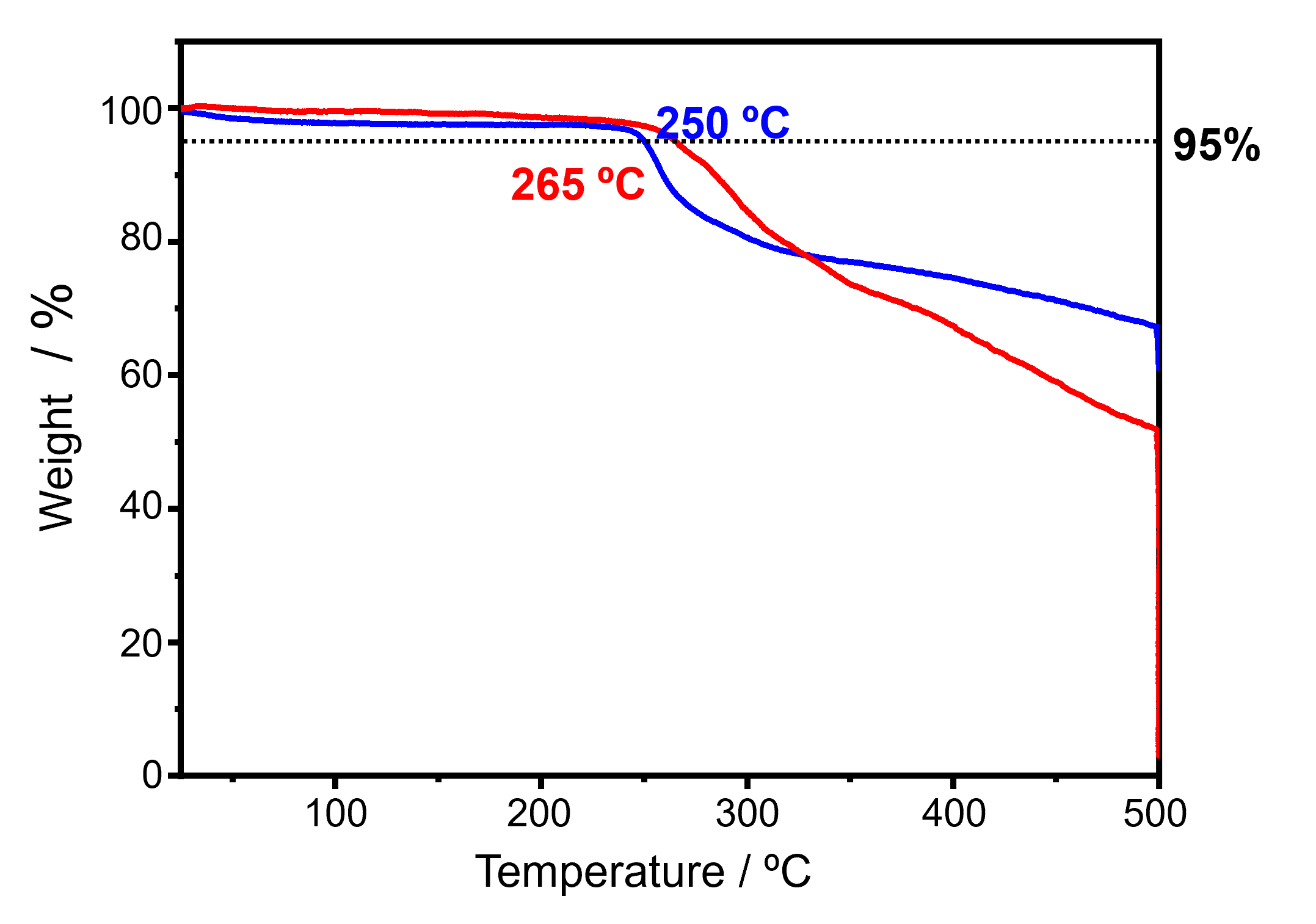


**Figure S1**. TGA curves of **Py-FNTz-B** (blue) and **IP-FNTz-B** (red).


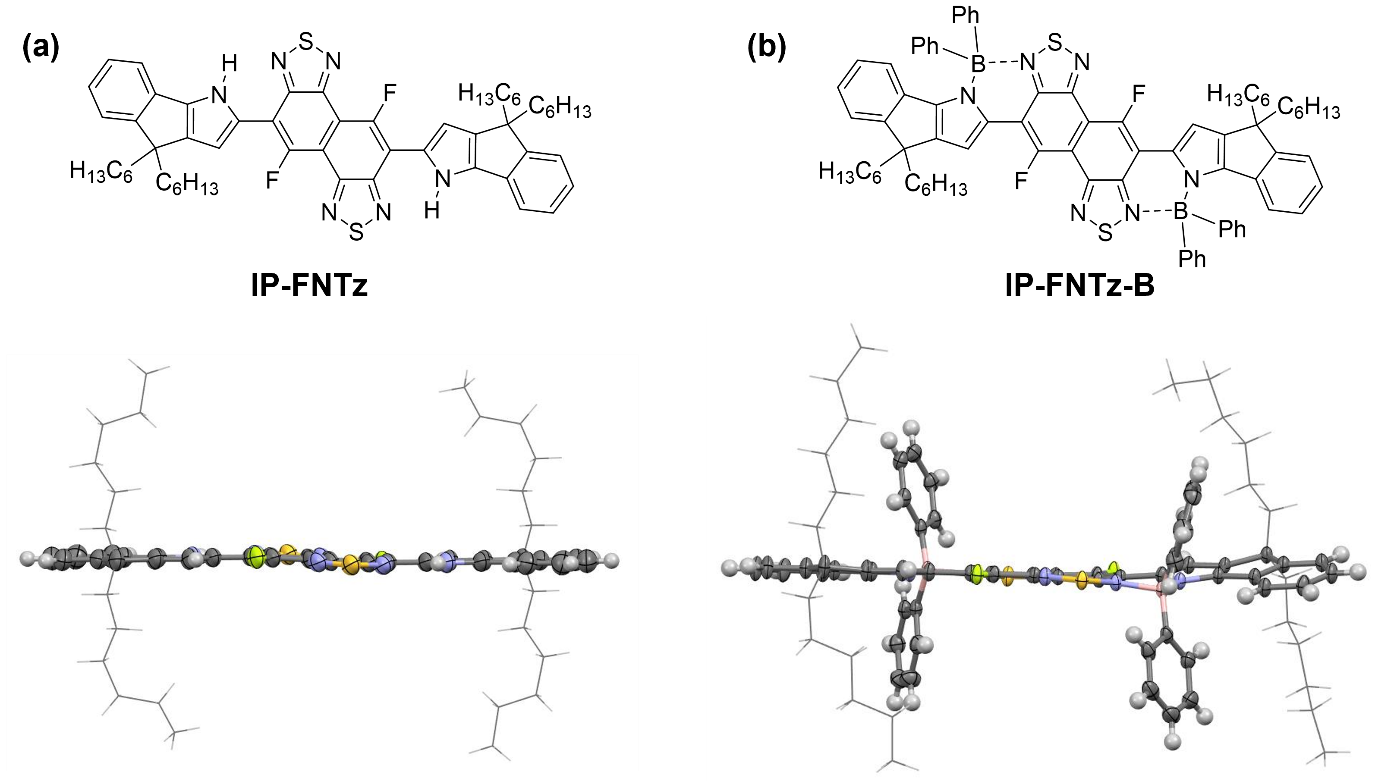


**Figure S2**. Side views of the single-crystal structures of (a) **IP-FNTz** and (b) **IP-FNTz-B**.


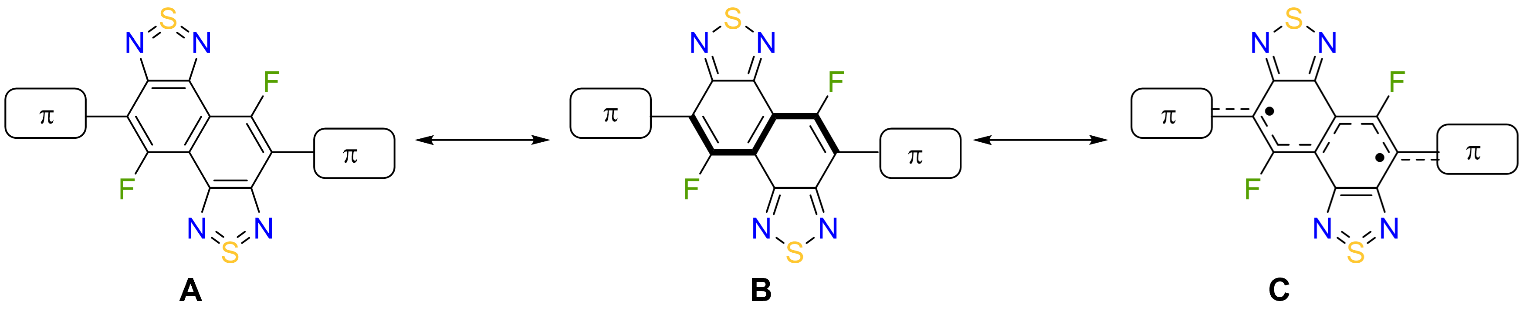


**Figure S3**. Resonance structures of the FNTz-based π-conjugated molecule.


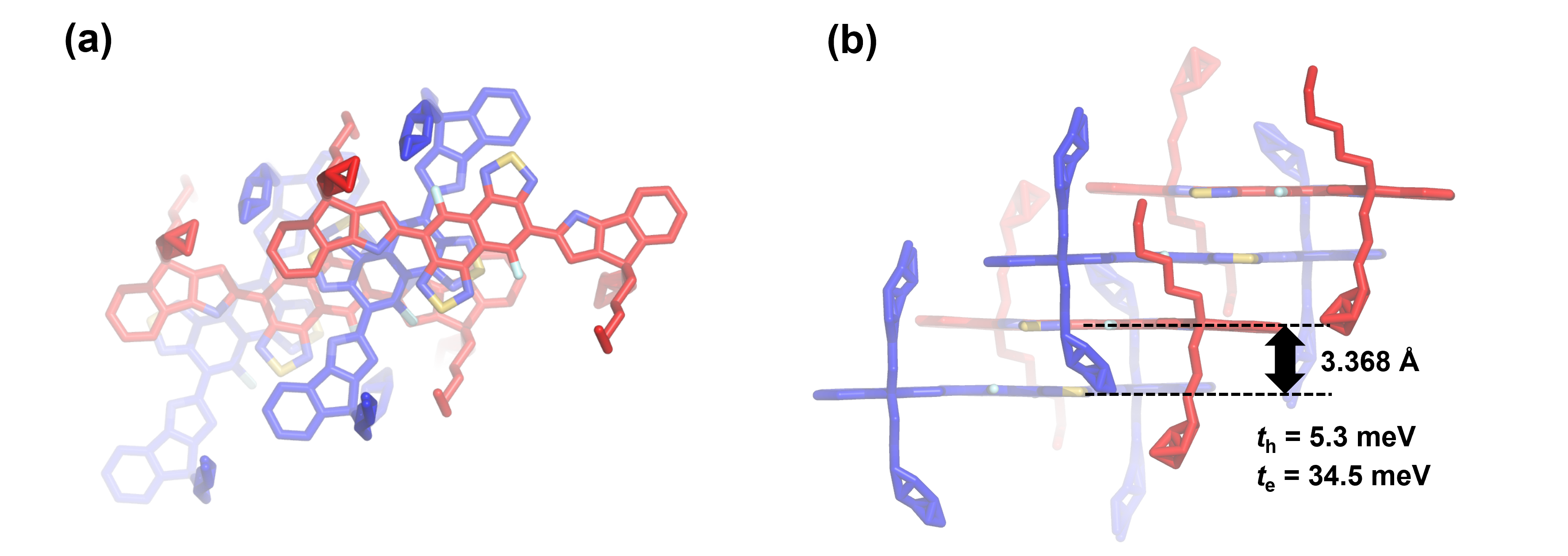


**Figure S4**. (a) Top and (b) side views of the packing diagram for **IP-FNTz**.


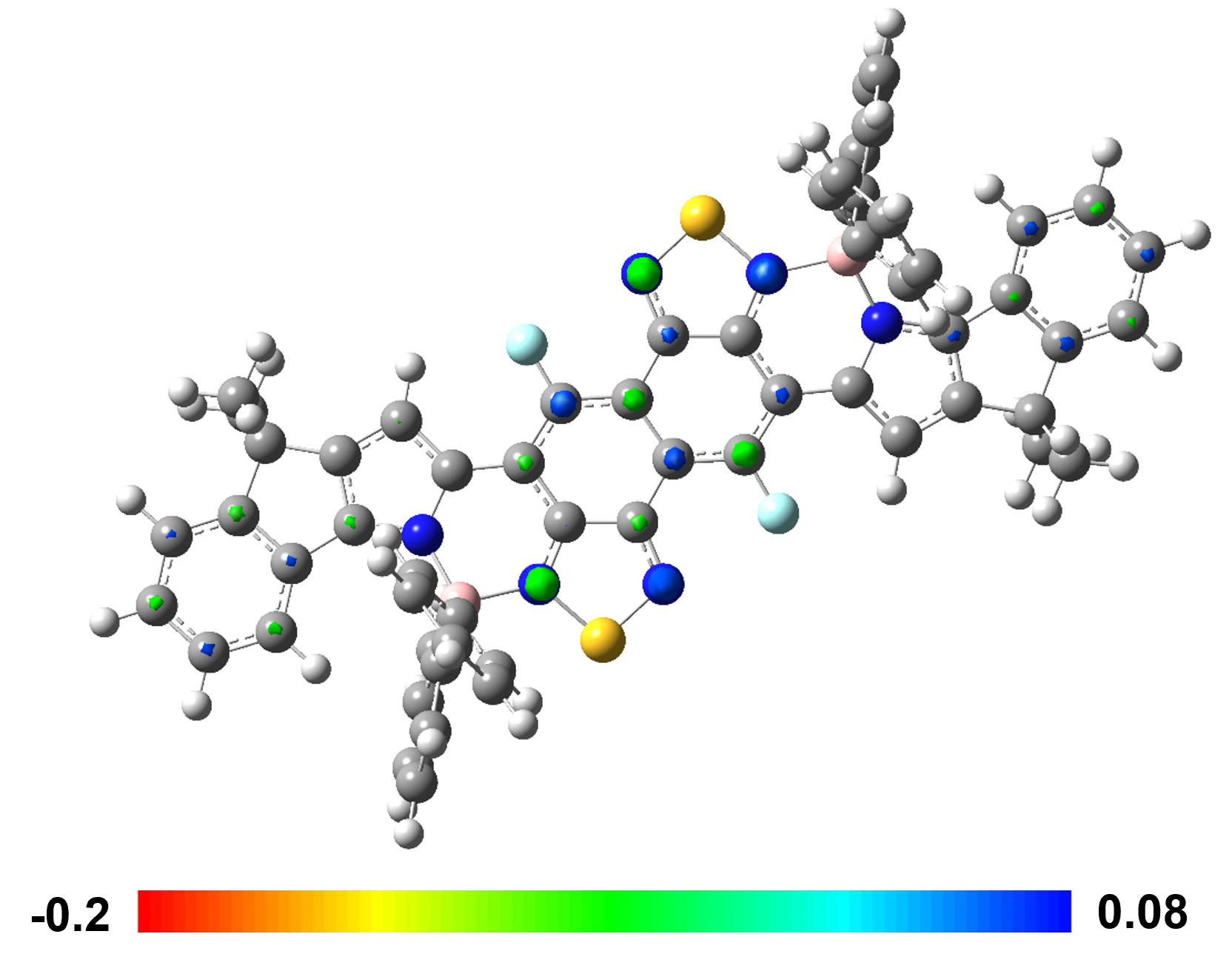


**Figure S5**. Spin density mapping of **FNTz-IP-B**’ at UHF/6-31g(d,p). Isovalue for surfaces is set to MO = 0.06 and density = 0.0004.


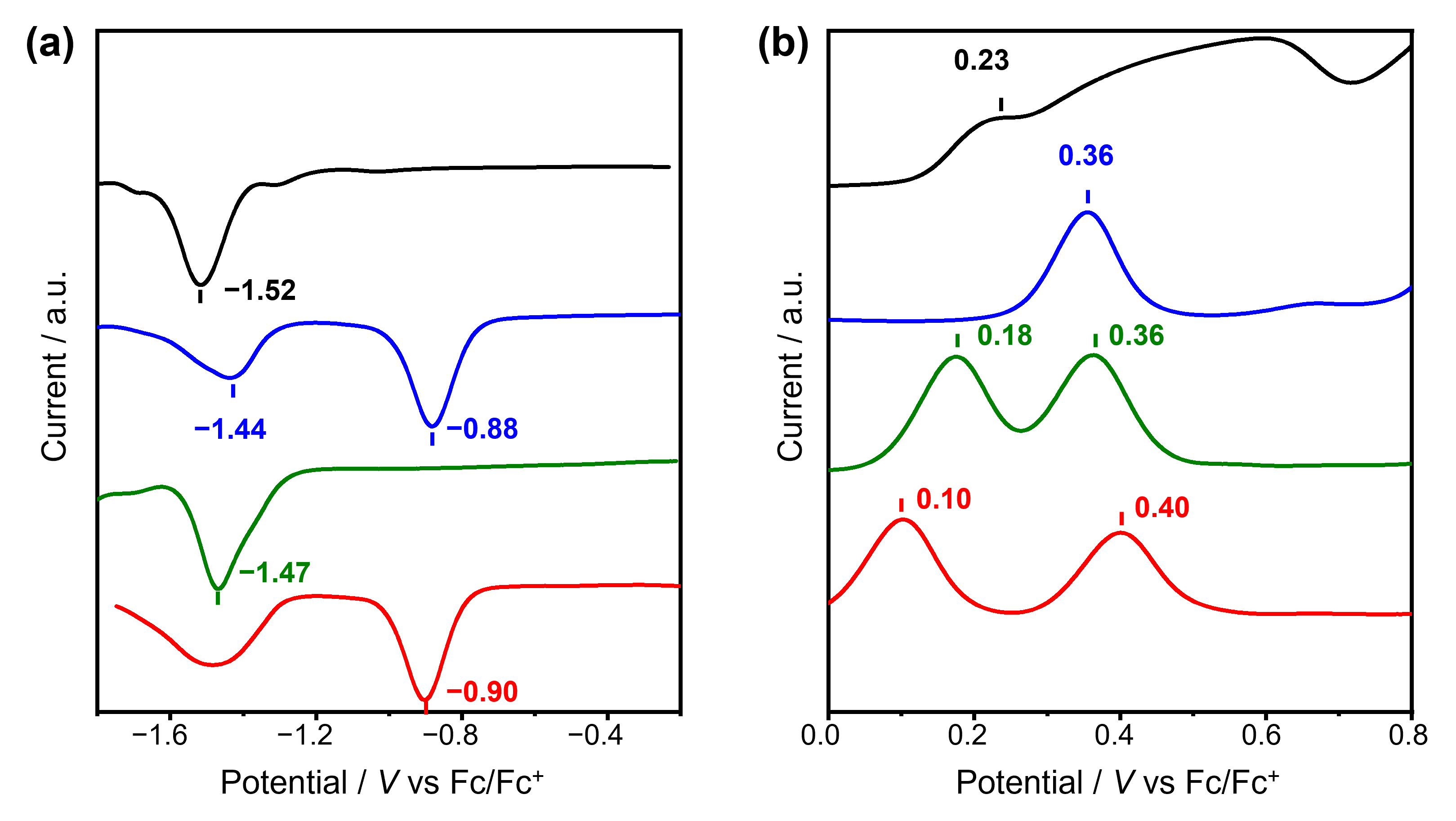


**Figure S6.** Differential pulse voltammograms for **Py-FNTz** (black), **Py-FNTz-B** (red), **IP-FNTz** (blue), and **IP-FNTz-B** (green) in dichloromethane containing 0.1 m of TBAPF_6_.


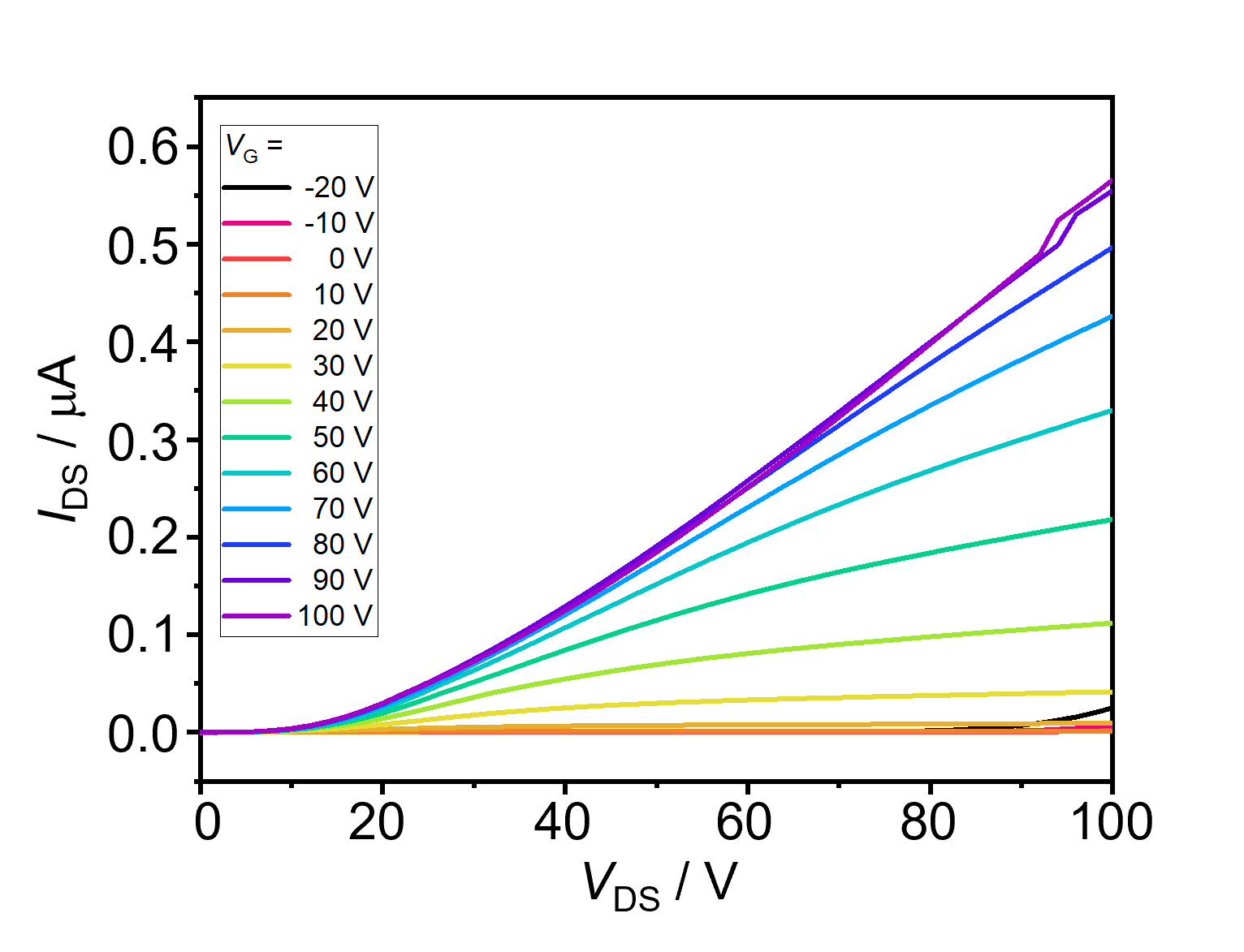


**Figure S7**. Output characteristics for OFET based on **Py-FNTz-B**.


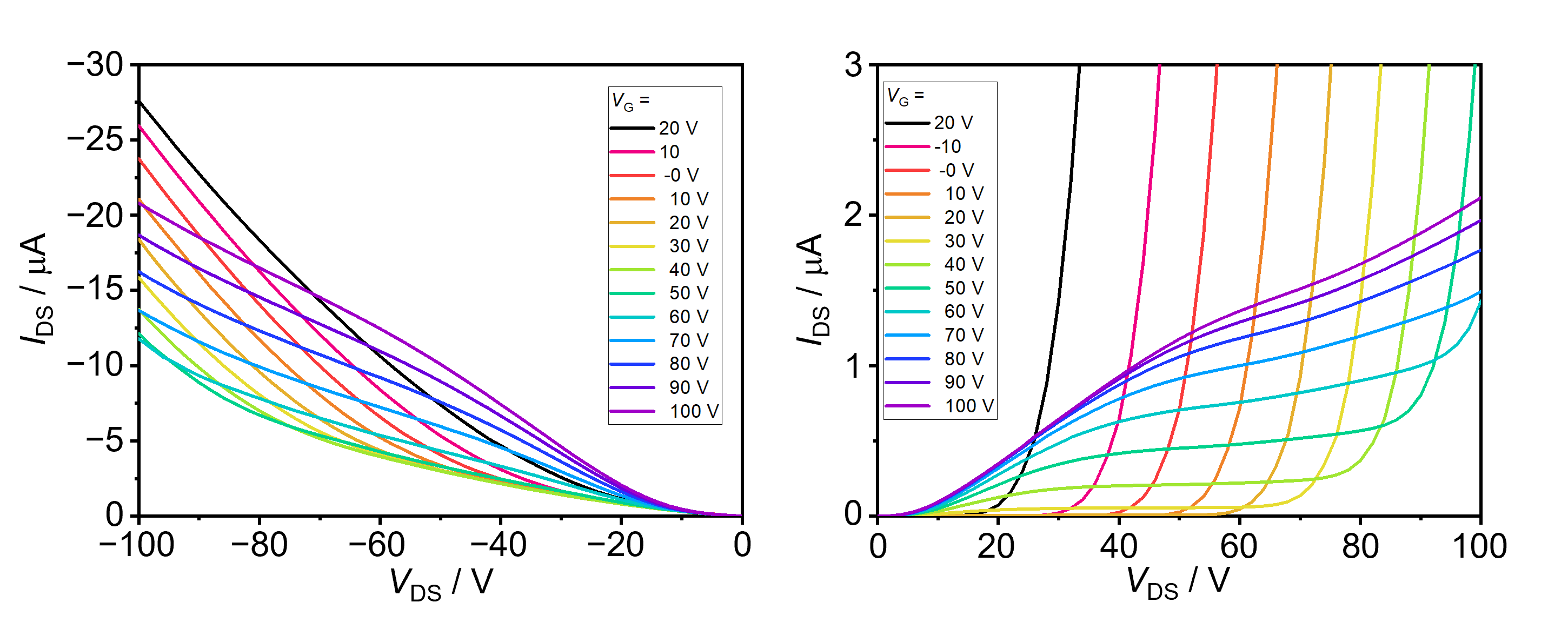


**Figure S8**. Output characteristics for OFET based on **IP-FNTz-B**.

**Table S1**. OFET characteristics for **Py-FNTz-B**.

| Entry | Conc.  (wt%) | Solvent*^a^* | Surface | Annealing | μ_e_ [cm^2^ V^−1^ s^−1^] | *V*_th_ [V] |
| --- | --- | --- | --- | --- | --- | --- |
| 1 | 0.25 | CF | Bare | 180 °C | 1.2 × 10^−7^ | 20 |
| 2 | 0.25 | Hot CF*^b^* | Bare | 180 °C | 1.2 × 10^−8^ | 3 |
| 3 | 0.25 | Hot CF*^b^* | ODTS-modified | 180 °C | 2.4 × 10^−5^ | 20 |
| 4 | 0.50 | Hot CF*^b^* | ODTS-modified | 180 °C | 1.4 × 10^−5^ | 11 |
| 5 | 1.0 | CF | ODTS-modified | 150 °C | 4.1 × 10^−7^ | 12 |
| 6 | 1.0 | Hot CF*^b^* | ODTS-modified | 180 °C | 2.3 × 10^−5^ | 29 |
| 7 | 1.0 | Hot CB*^c^* | ODTS-modified | w/o | 6.0 × 10^−7^ | 28 |

[a] CF and CB means chloroform and chlorobenzene, respectively. [b] Warmed at 60 °C. [c] Warmed at 120 °C.

**Table S2**. OFET characteristics for **IP-FNTz-B**. The active layer was prepared by spin-coating (1 wt% solution in CHCl_3_, 1500 rpm) onto an ODTS-modified SiO_2_/Si substrate.

| Annealing | μ_h_ [cm^2^ V^−1^ s^−1^] | μ_e_ [cm^2^ V^−1^ s^−1^] | V_th_ [V] for hole | V_th_[V] for electron |
| --- | --- | --- | --- | --- |
| w/o | 2.0 × 10^−4^ | 9.5 × 10^−5^ | −11 | 20 |
| 150 °C | 4.1 × 10^−5^ | 3.1 × 10^−5^ | −24 | 29 |
| 180 °C | No FET | 5.4 × 10^−7^ | No FET | 45 |


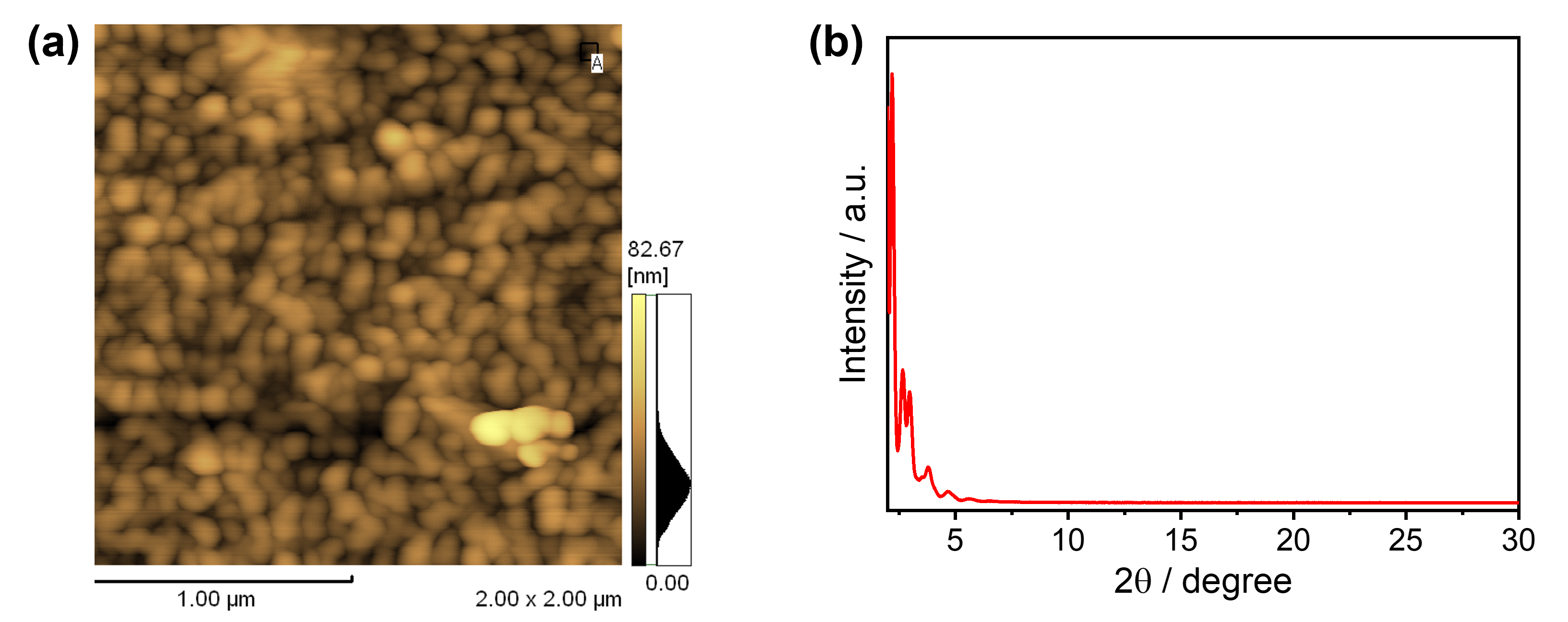


**Figure S9.** (a) AFM image and (b) X-ray diffraction patterns for OFET based on **Py-FNTz-B**.


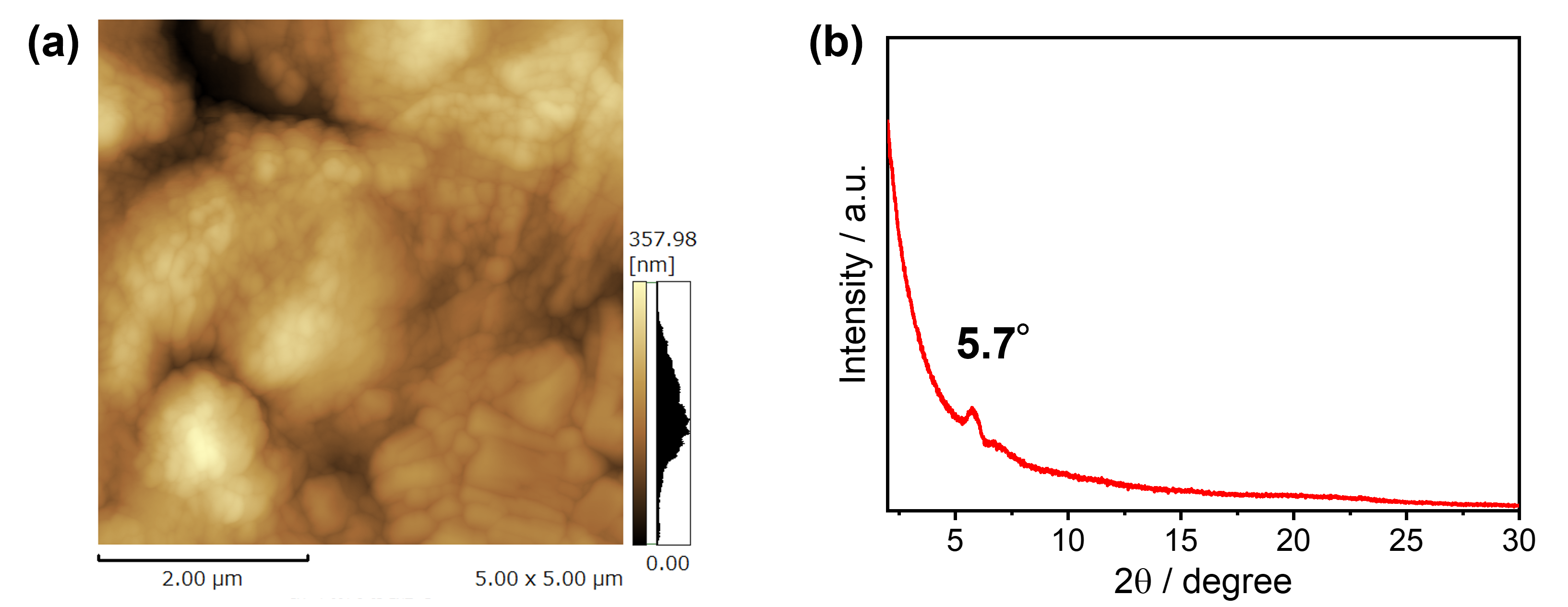


**Figure S10.** (a) AFM image and (b) X-ray diffraction patterns for OFET based on **IP-FNTz-B**.


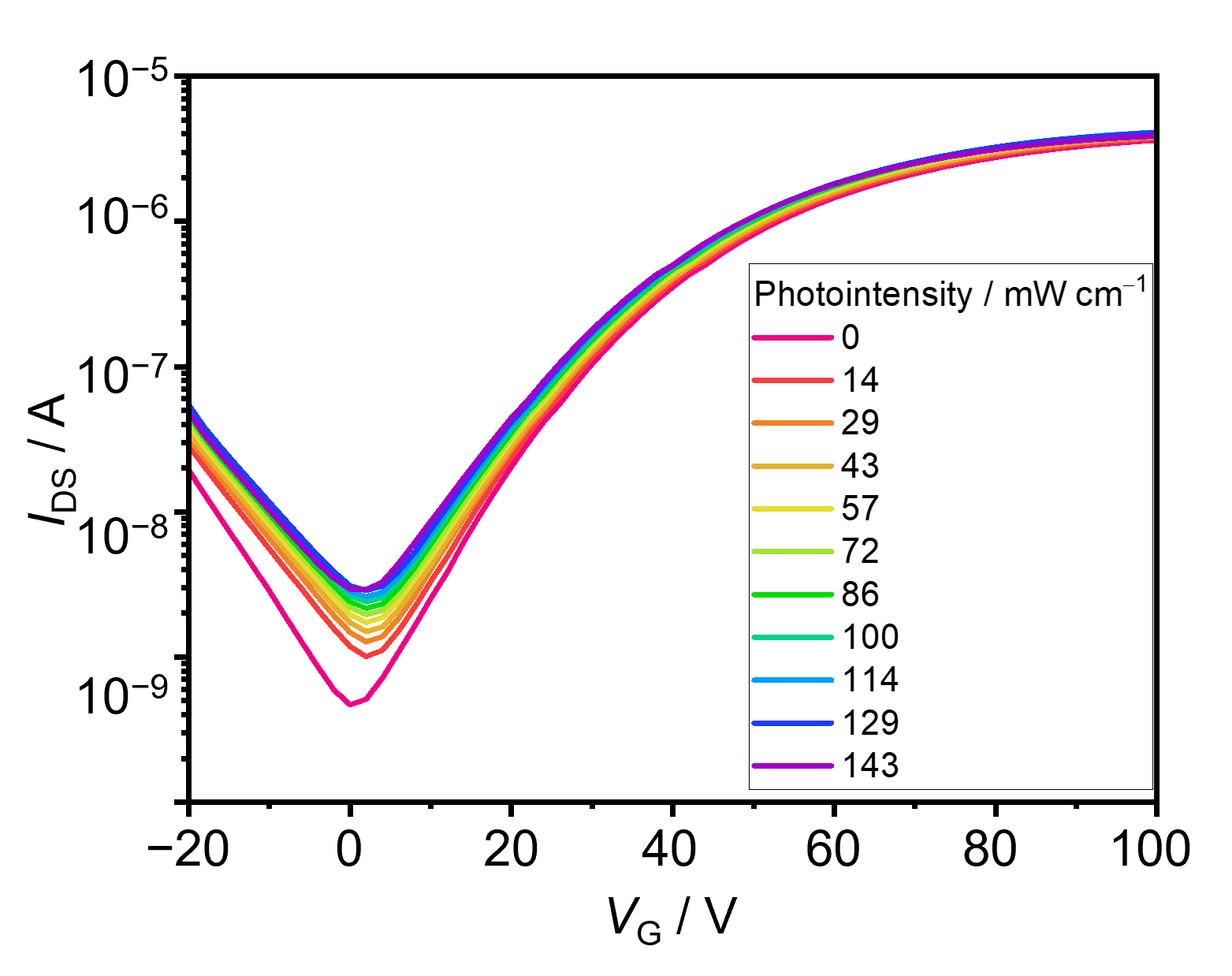


**Figure S11**. Photointensity dependence on transfer characteristics.


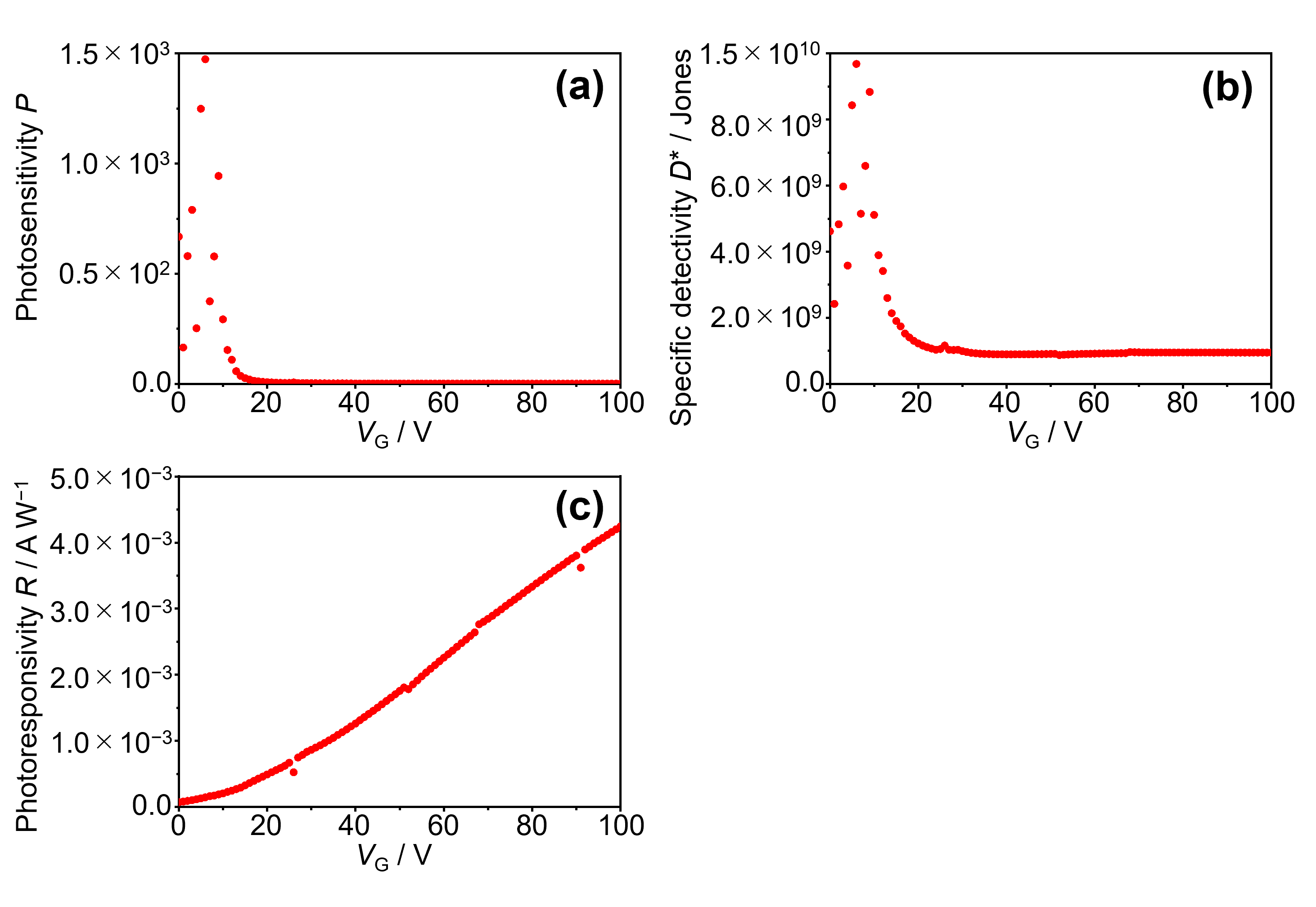


**Figure S12**. (a) photosensitivity (*P*), (b) specific detectivity (*D**), and (c) photoresponsivity (*R*) as a function of *V*_G_.


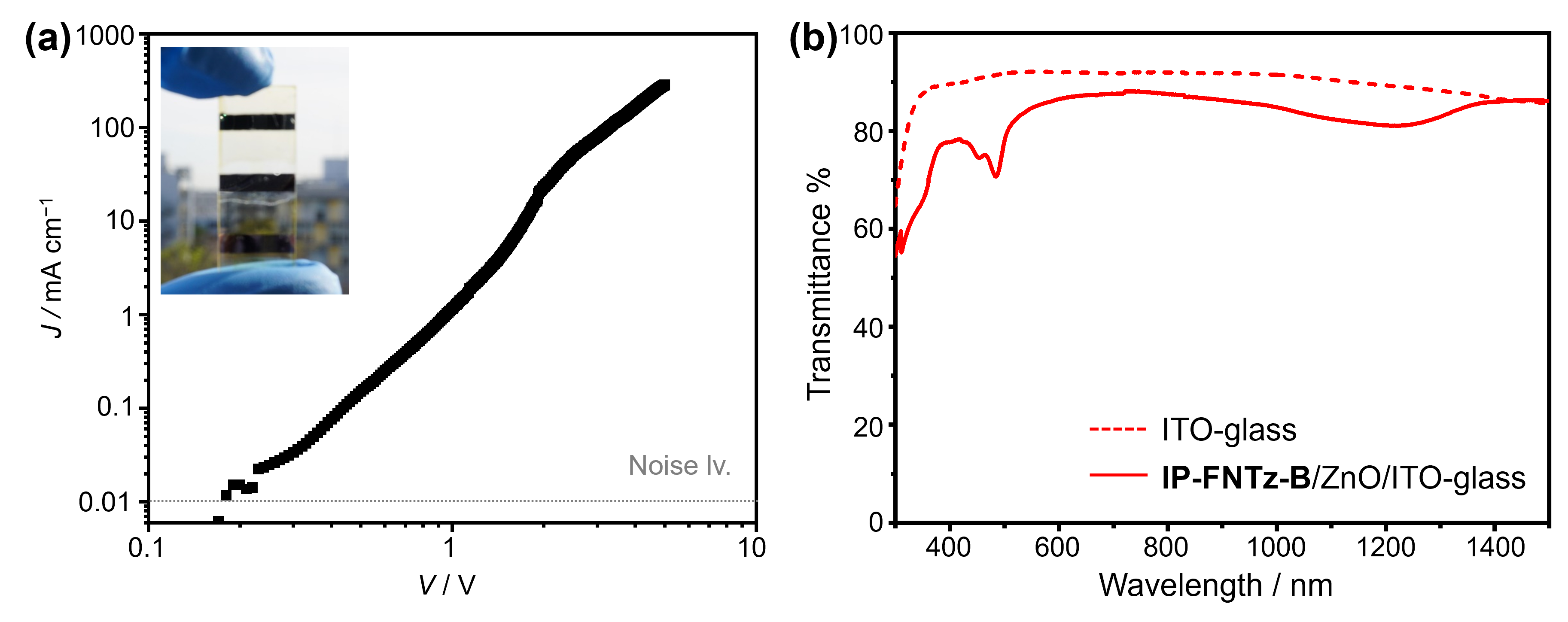


**Figure S13**. (a) *J*–*V* plots for a hole-only device of ITO/ZnO/**IP-FNTz-B**/Ca/Al. Inset shows a photograph of the device. (b) Transmittance spectra of ITO-glass (broken line) and **IP-FNTz-B**/ZnO/ITO-glass.


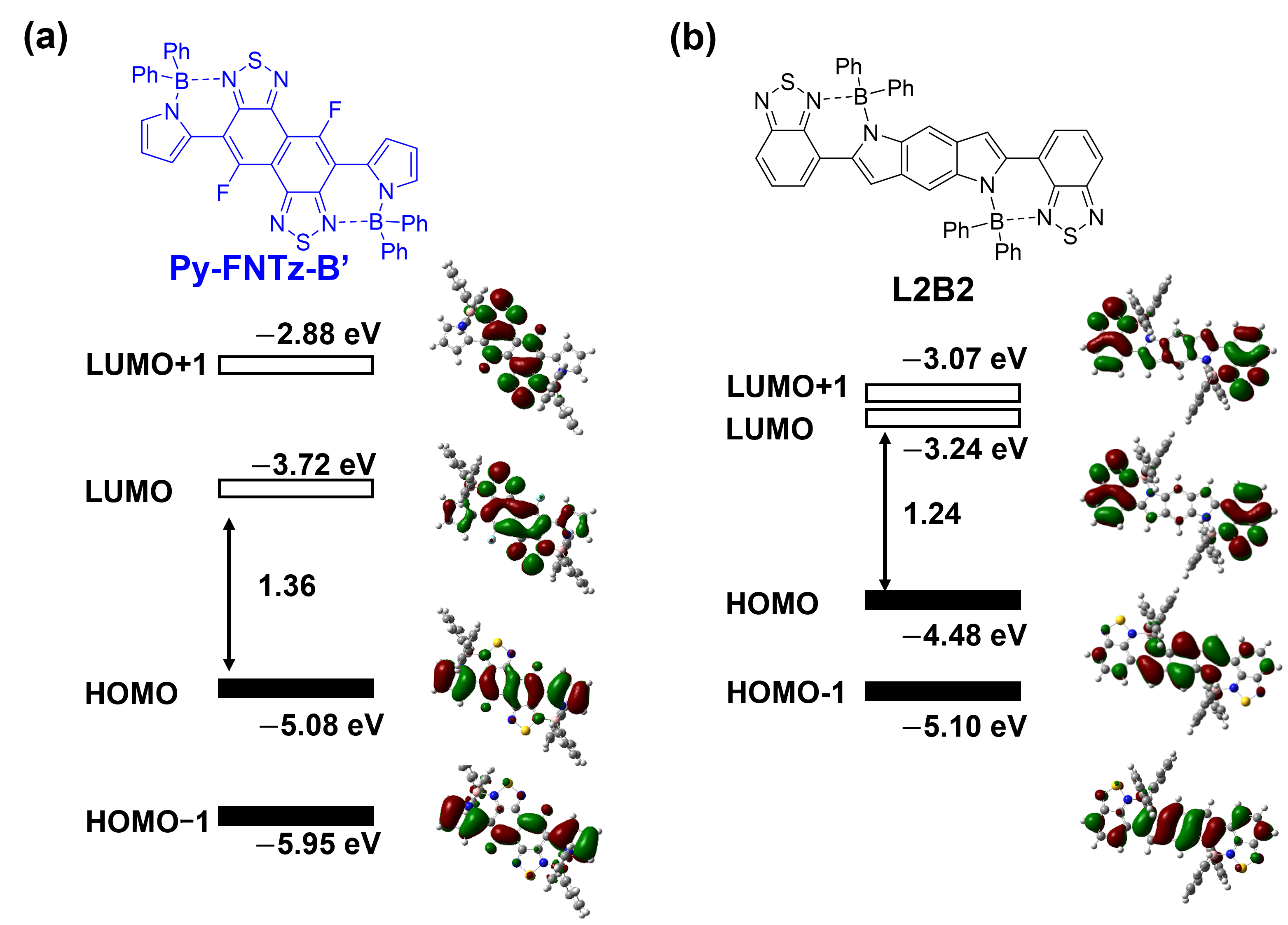


**Figure 14.** Energy diagrams of the Kohn-Sham orbitals of (a) **Py-FNTz-B’** and (b) **L2B2**.

Our molecular design also allows us to rationalize the transparency in the reported compounds. For example, BODIPY-1 has a polyene structure in the entire π-skeleton, resulting in almost exclusive NIR absorption, as shown in Figure S15. BODIPY-2 exhibits a similar trend. In contrast, BODIPY-3, despite its structural similarity to BODIPY-2, has less contribution from polyene structures, resulting in strong absorption in visible light and a colored solution.


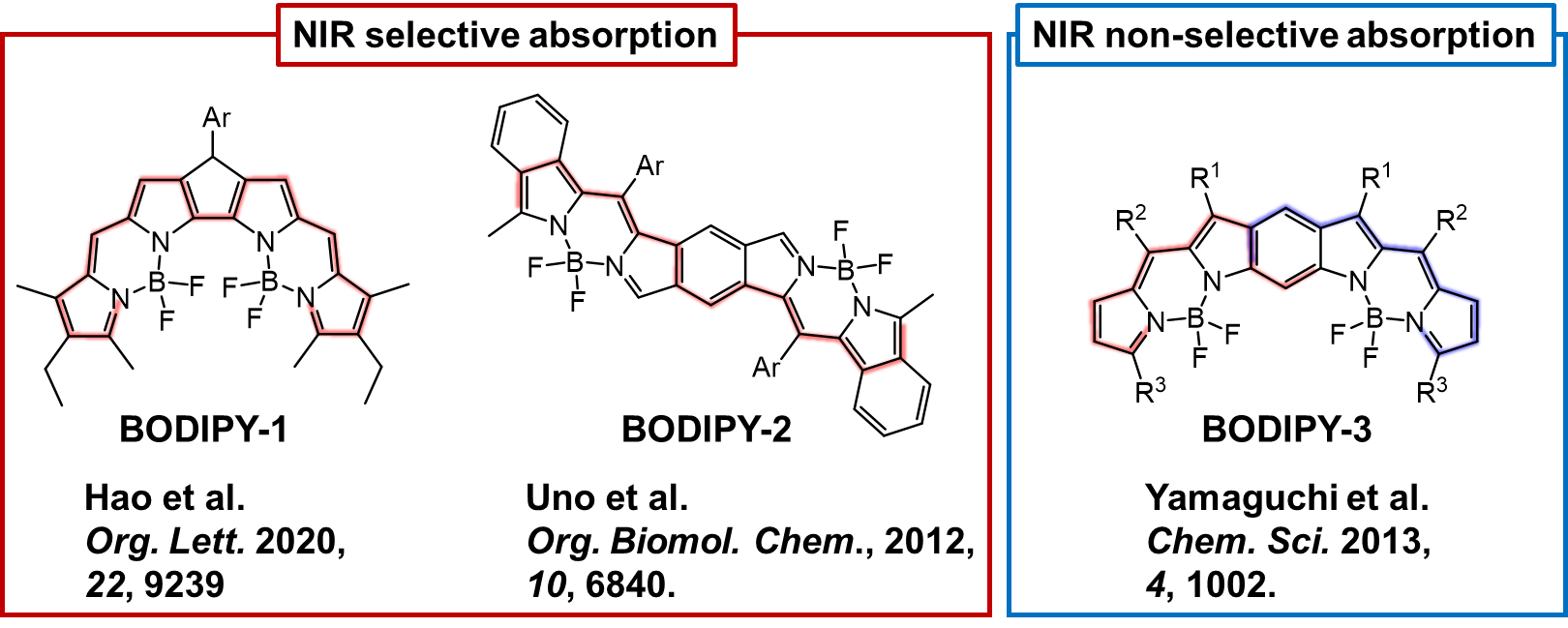


**Figure S15**. Molecular structures of BODIPY-1, BODIPY-2, and BODIPY-3. Red and blue lines on molecular structures denote the polyene π-conjugations.

**X-ray Crystal Analysis**

Diffraction data were collected on a Rigaku Oxford Diffraction XtaLAB PRO diffractometer with CuKα radiation (λ = 1.54187 Å). The single crystal of **IP-FNTz** and **IP-FNTz-B** was obtained by recrystallization from chlorobenzene/methanol. The X-ray measurements were performed at −170 °C. The structures were solved by direct methods (SHELXT) and refined through full-matrix least-squares techniques on F2 using SHELXL and OLEX2 crystallographic software packages.^[S6]-[S8]^ All non-hydrogen atoms were refined with anisotropic displacement parameters, and hydrogen atoms were placed at calculated positions and refined “riding” on their corresponding carbon atoms. Crystallographic data are available from the Supporting Information or from The Cambridge Crystallographic Data Centre, codes 2308994 for **IP-FNTz** and 2308995 for **IP-FNTz-B.**


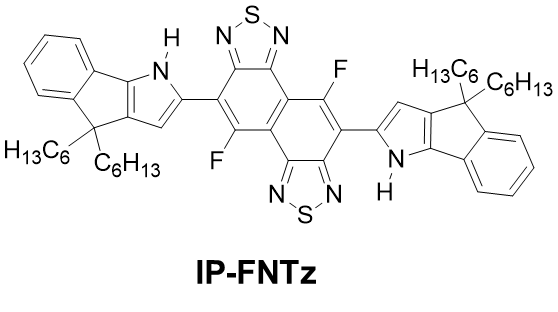


| Compound | **IP-FNTz (**CCDC: 2308994**)** |
| --- | --- |
| Empirical formula | C_37.33_H_42.67_F_1.33_N_4_S_1.33_ |
| Formula weight | 615.50 |
| Temperature/K | 103.15 |
| Crystal system | triclinic |
| Space group | P-1 |
| a/Å | 10.9895(2) |
| b/Å | 16.5502(2) |
| c/Å | 16.7953(2) |
| α/° | 103.0750(10) |
| β/° | 97.8310(10) |
| γ/° | 90.3110(10) |
| Volume/Å^3^ | 2945.68(7) |
| Z | 3 |
| ρ_calc_g/cm^3^ | 1.041 |
| μ/mm^‑1^ | 1.158 |
| F(000) | 984.0 |
| Crystal size/mm^3^ | 0.563 × 0.119 × 0.105 |
| Radiation | Cu Kα (λ = 1.54184) |
| 2Θ range for data collection/° | 5.456 to 136.5 |
| Index ranges | -10 ≤ h ≤ 13, -19 ≤ k ≤ 19, -20 ≤ l ≤ 20 |
| Reflections collected | 34559 |
| Independent reflections | 10740 [R_int_ = 0.0278, R_sigma_ = 0.0283] |
| Data/restraints/parameters | 10740/0/653 |
| Goodness-of-fit on F^2^ | 1.066 |
| Final R indexes [I>=2σ (I)] | R_1_ = 0.0543, wR_2_ = 0.1635 |
| Final R indexes [all data] | R_1_ = 0.0601, wR_2_ = 0.1690 |
| Largest diff. peak/hole / e Å^-3^ | 0.31/-0.28 |


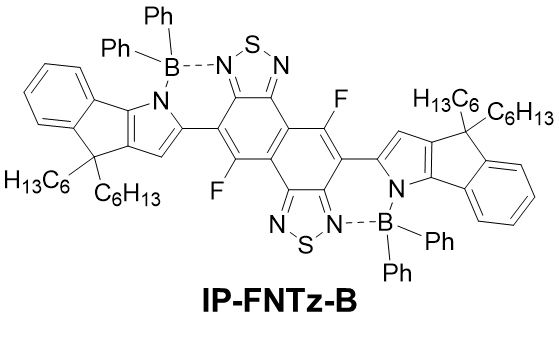


| Compound | **IP-FNTz-B** (CCDC: 2308995**)** |
| --- | --- |
| Empirical formula | C_92_H_92_B_2_Cl_2_F_2_N_6_S_2_ |
| Formula weight | 1476.35 |
| Temperature/K | 103.15 |
| Crystal system | monoclinic |
| Space group | C2/c |
| a/Å | 29.6700(3) |
| b/Å | 19.4995(2) |
| c/Å | 28.9708(3) |
| α/° | 90 |
| β/° | 106.7840(10) |
| γ/° | 90 |
| Volume/Å^3^ | 16047.0(3) |
| Z | 8 |
| ρ_calc_g/cm^3^ | 1.222 |
| μ/mm^‑1^ | 1.639 |
| F(000) | 6240.0 |
| Crystal size/mm^3^ | 0.459 × 0.269 × 0.183 |
| Radiation | Cu Kα (λ = 1.54184) |
| 2Θ range for data collection/° | 5.498 to 136.502 |
| Index ranges | -35 ≤ h ≤ 35, -23 ≤ k ≤ 23, -32 ≤ l ≤ 34 |
| Reflections collected | 49519 |
| Independent reflections | 14659 [R_int_ = 0.0412, R_sigma_ = 0.0367] |
| Data/restraints/parameters | 14659/15/988 |
| Goodness-of-fit on F^2^ | 1.034 |
| Final R indexes [I>=2σ (I)] | R_1_ = 0.0566, wR_2_ = 0.1607 |
| Final R indexes [all data] | R_1_ = 0.0607, wR_2_ = 0.1646 |
| Largest diff. peak/hole / e Å^-3^ | 0.87/-0.72 |

**NMR Spectra**


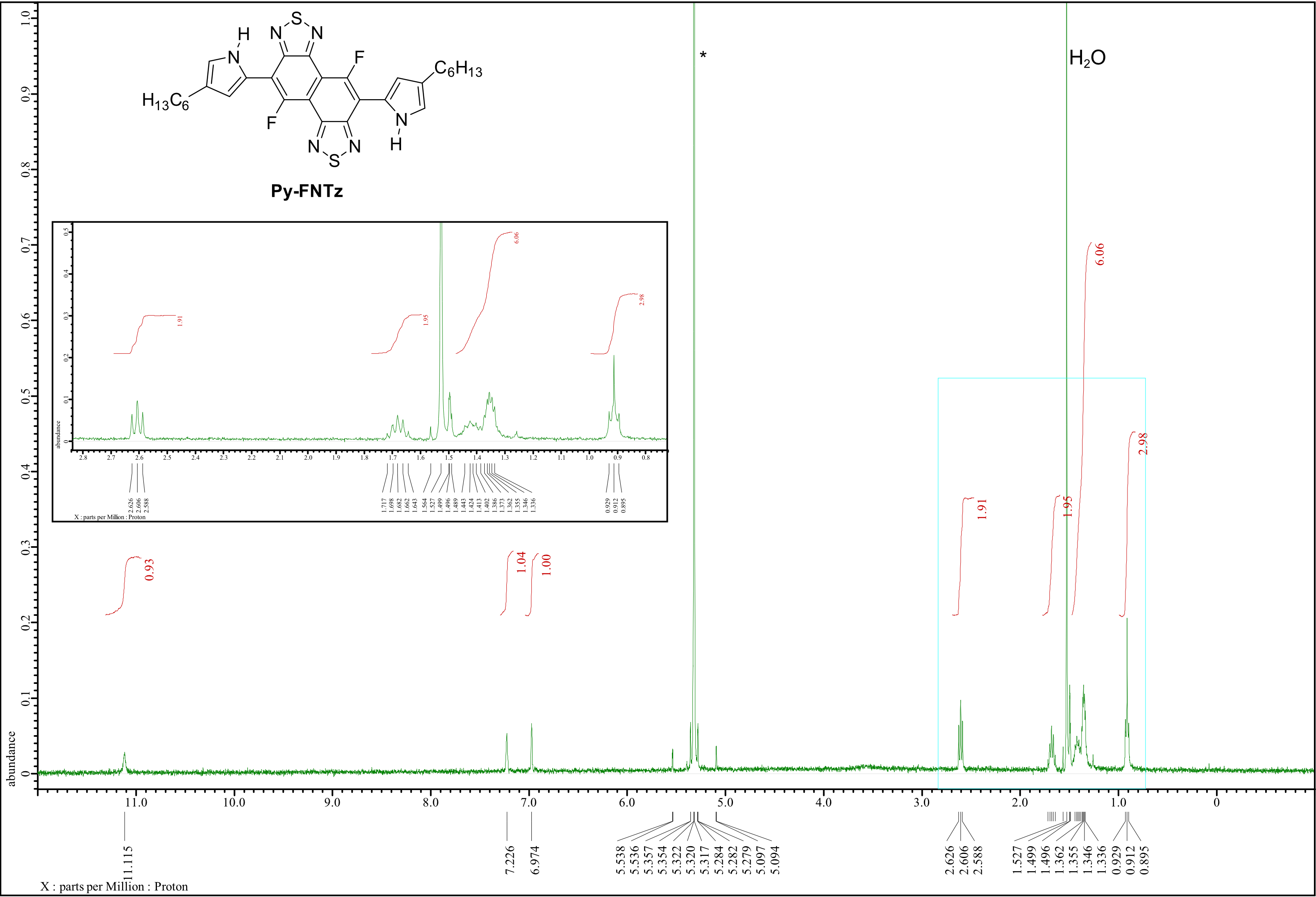


**Figure S16**. ^1^H NMR (400 MHz, dichloromethane-*d*_2_, r.t.) spectrum of **Py-FNTz**.


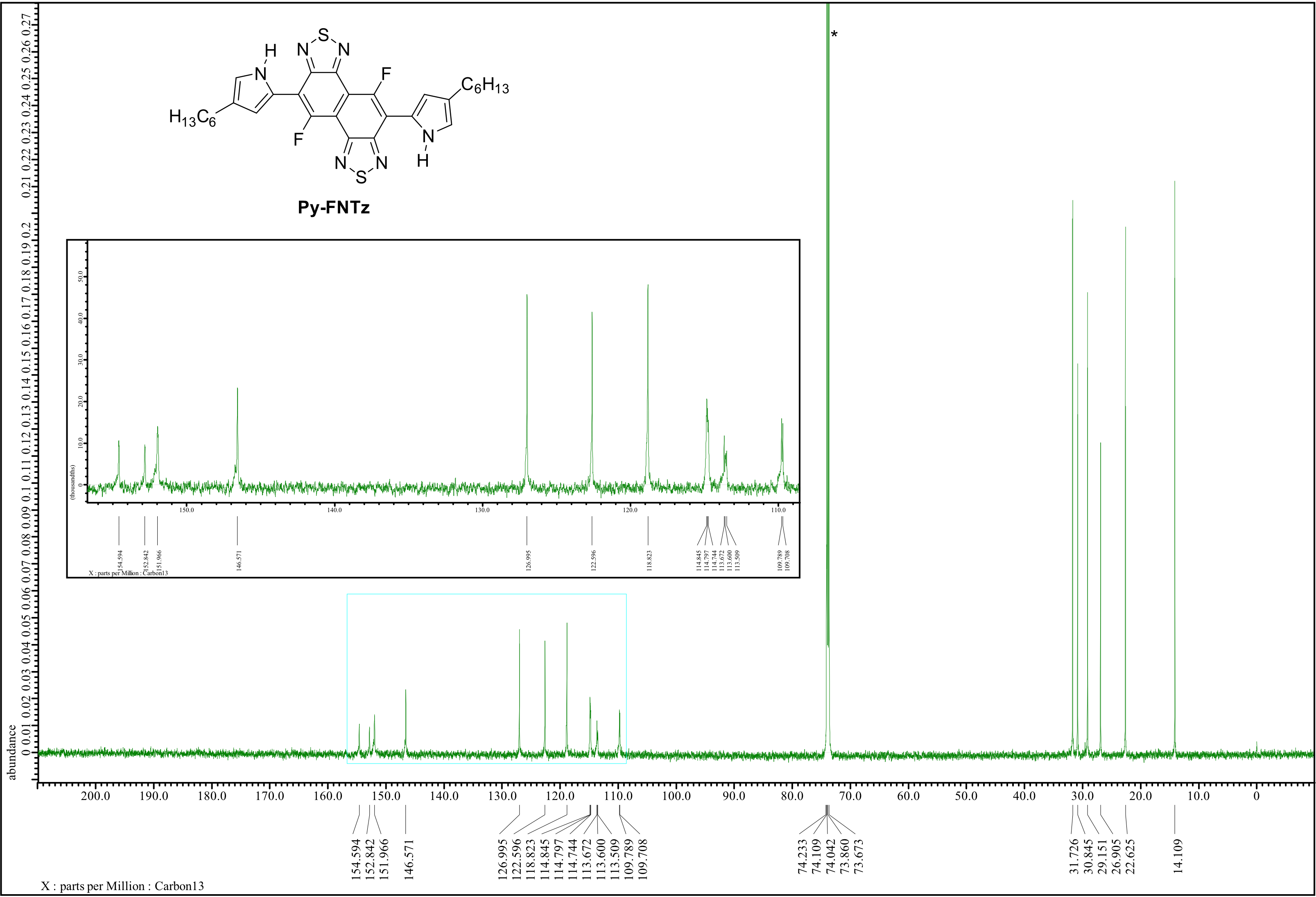


**Figure S17**. ^13^C NMR (151 MHz, tetrachloroethane-*d*_2_, 60 °C) spectrum of **Py-FNTz**. Asterisk denotes solvent peaks.


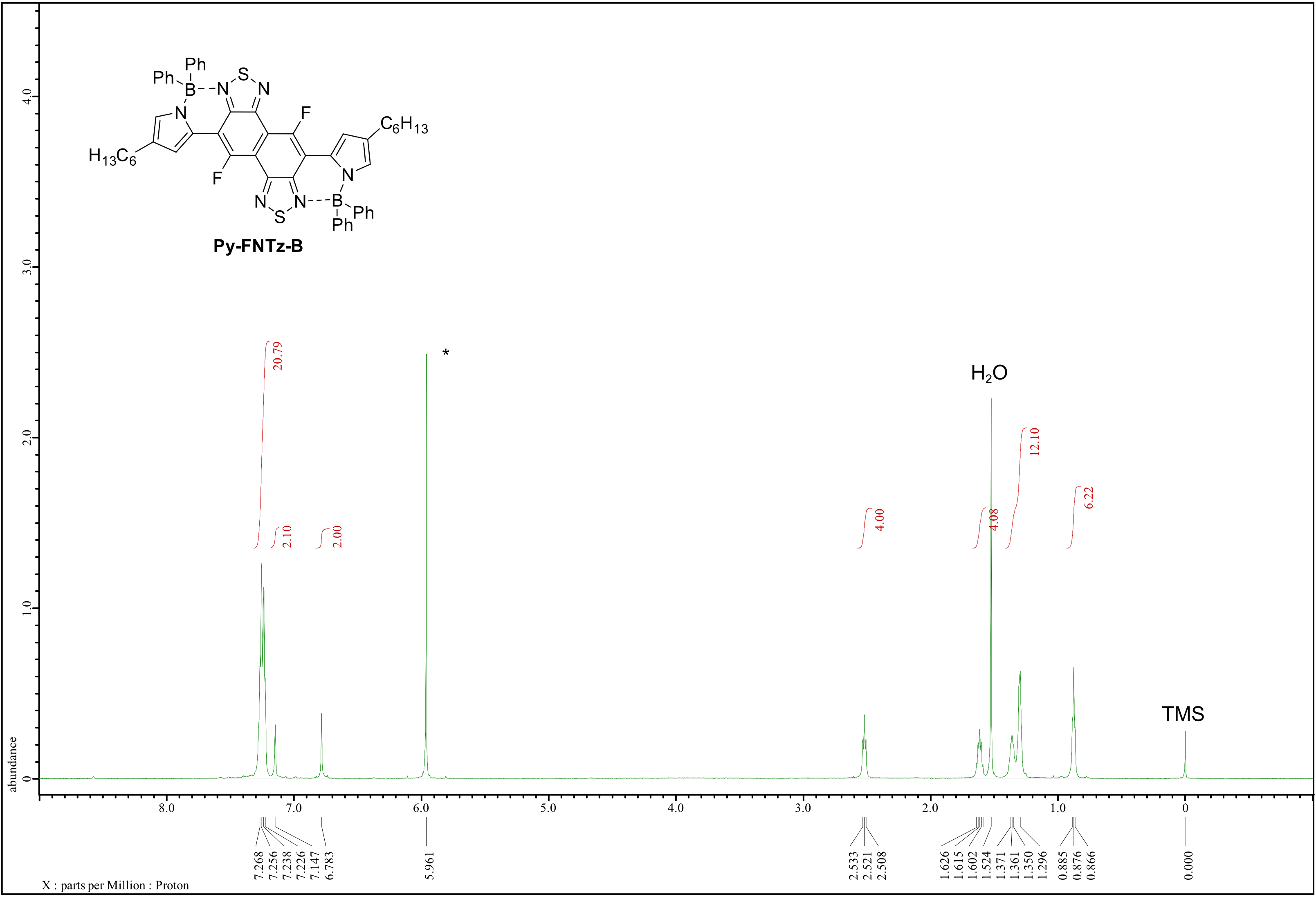


**Figure S18**. ^1^H NMR (600 MHz, tetrachloroethane-*d*_2_, 50 °C) spectrum of **Py-FNTz-B**. Asterisk denotes solvent peaks.


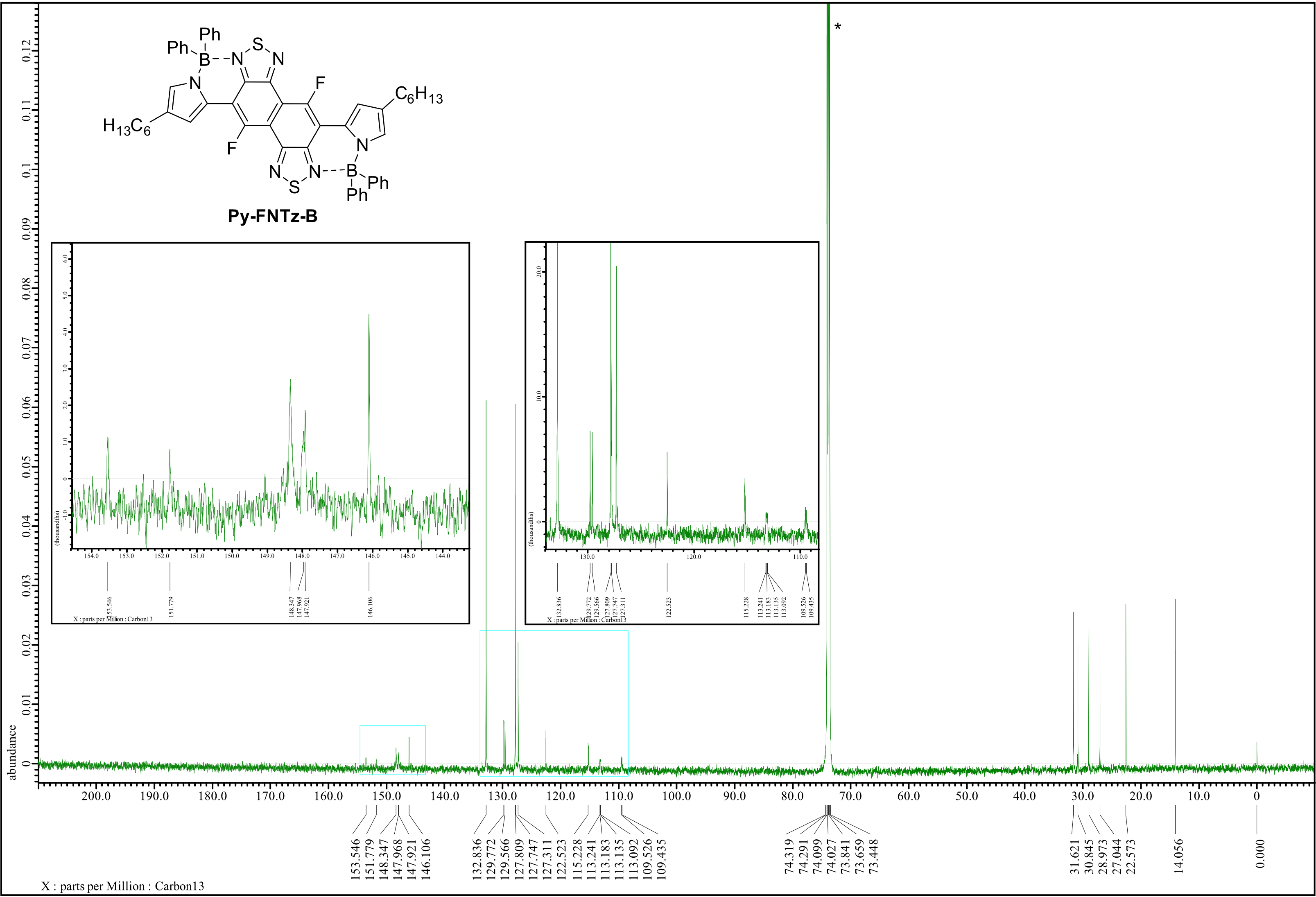


**Figure S19**. ^13^C NMR (101 MHz, tetrachloroethane-*d*_2_, 50 °C) spectrum of **Py-FNTz-B**. Asterisk denotes solvent peaks.


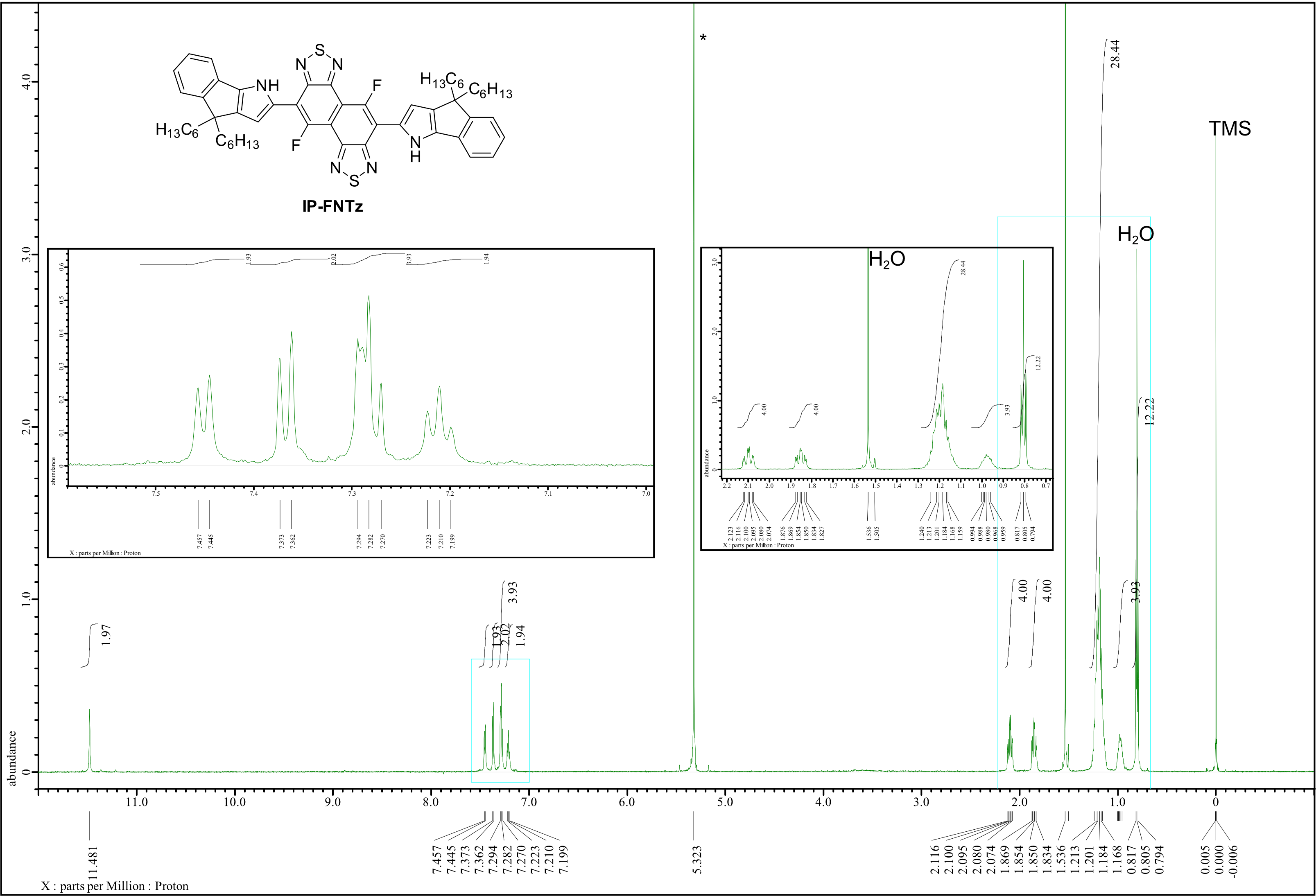


**Figure S20**. ^1^H NMR (600 MHz, dichloromethane-*d*_2_, r.t.) spectrum of **IP-FNTz**. Asterisk denotes solvent peaks.


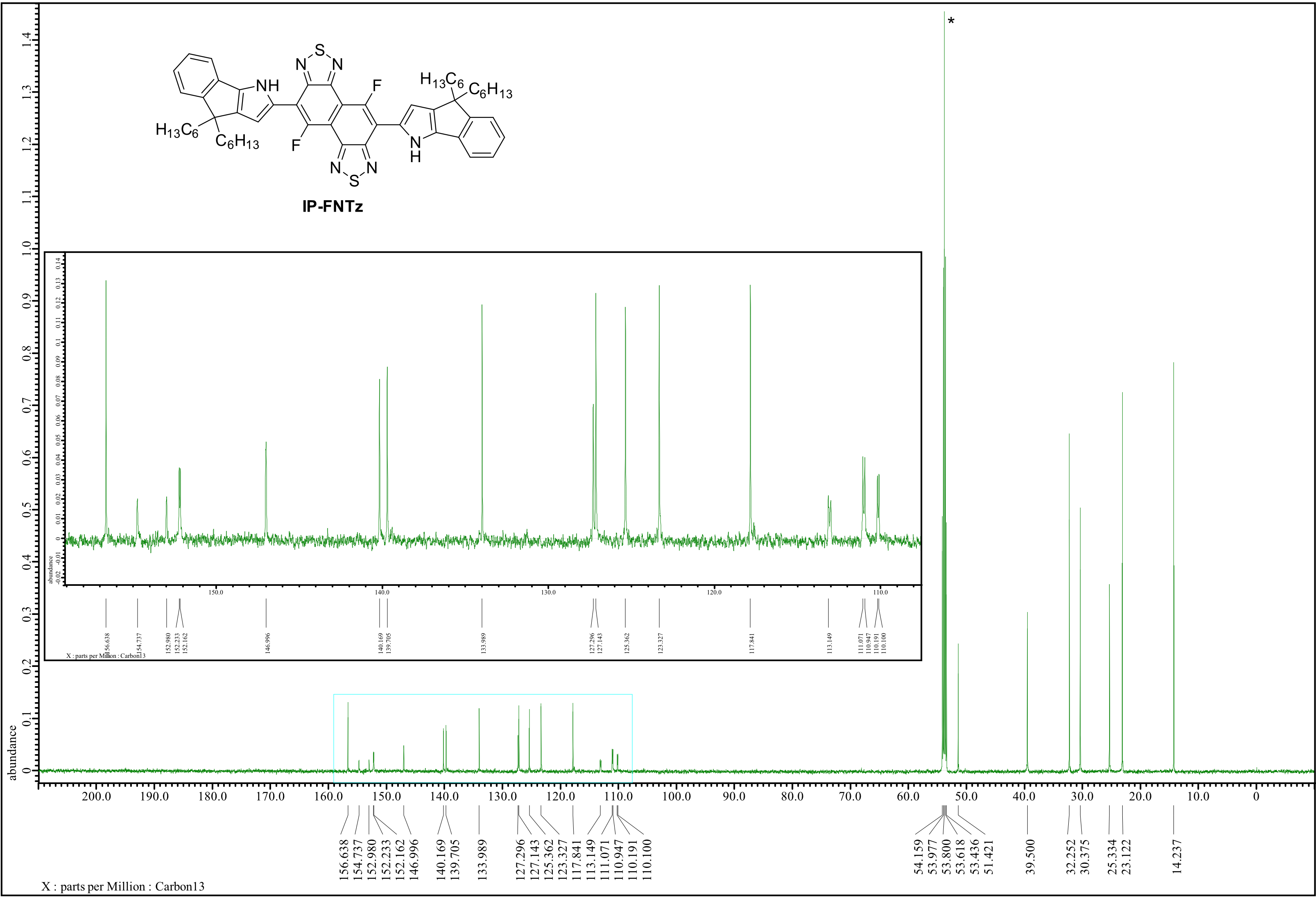


**Figure S21**. ^13^C NMR (151 MHz, dichloromethane-*d*_2_, r.t.) spectrum of **IP-FNTz**. Asterisk denotes solvent peaks.


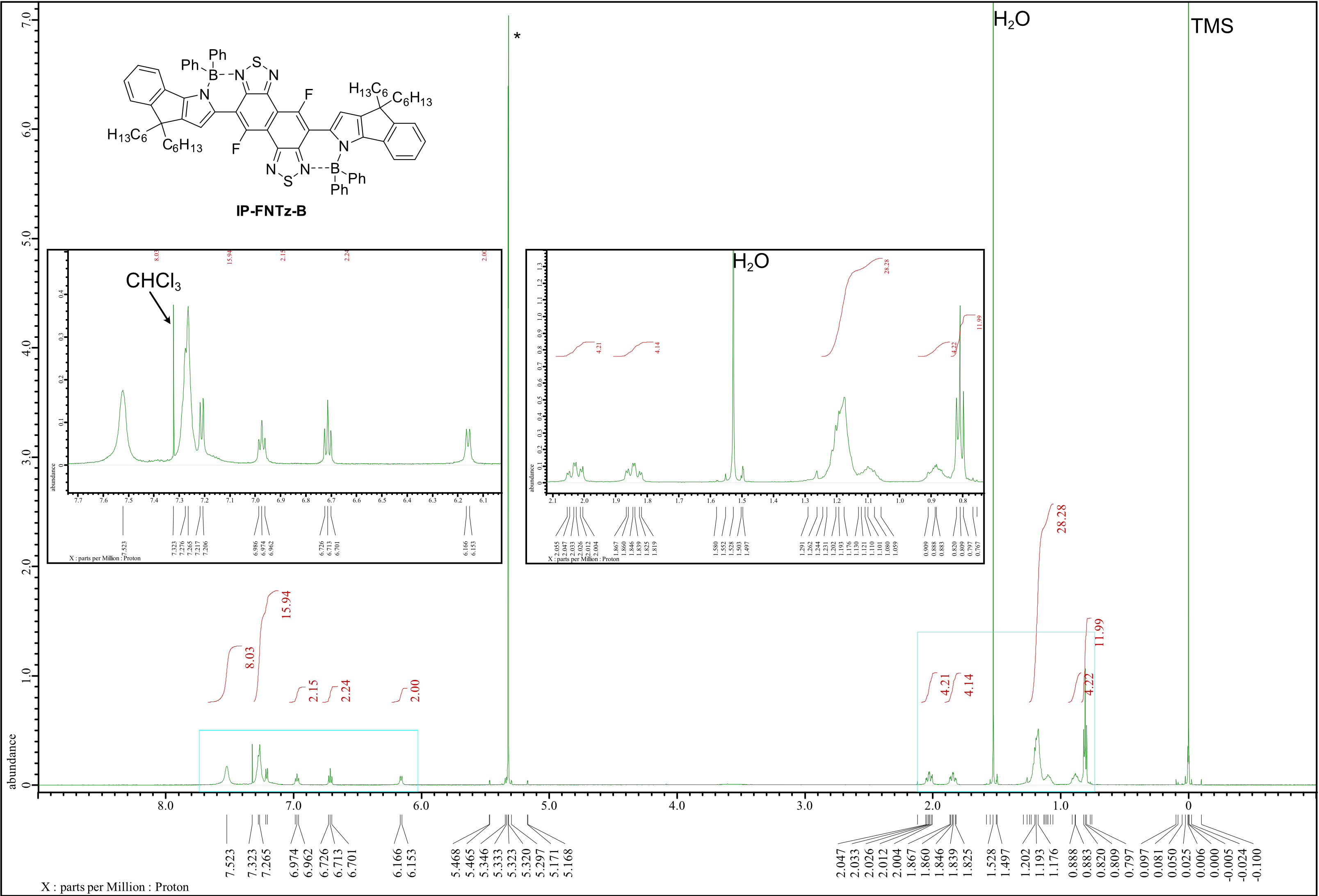


**Figure S22**. ^1^H NMR (600 MHz, dichloromethane-*d*_2_, r.t.) spectrum of **IP-FNTz-B**. Asterisk denotes solvent peaks.


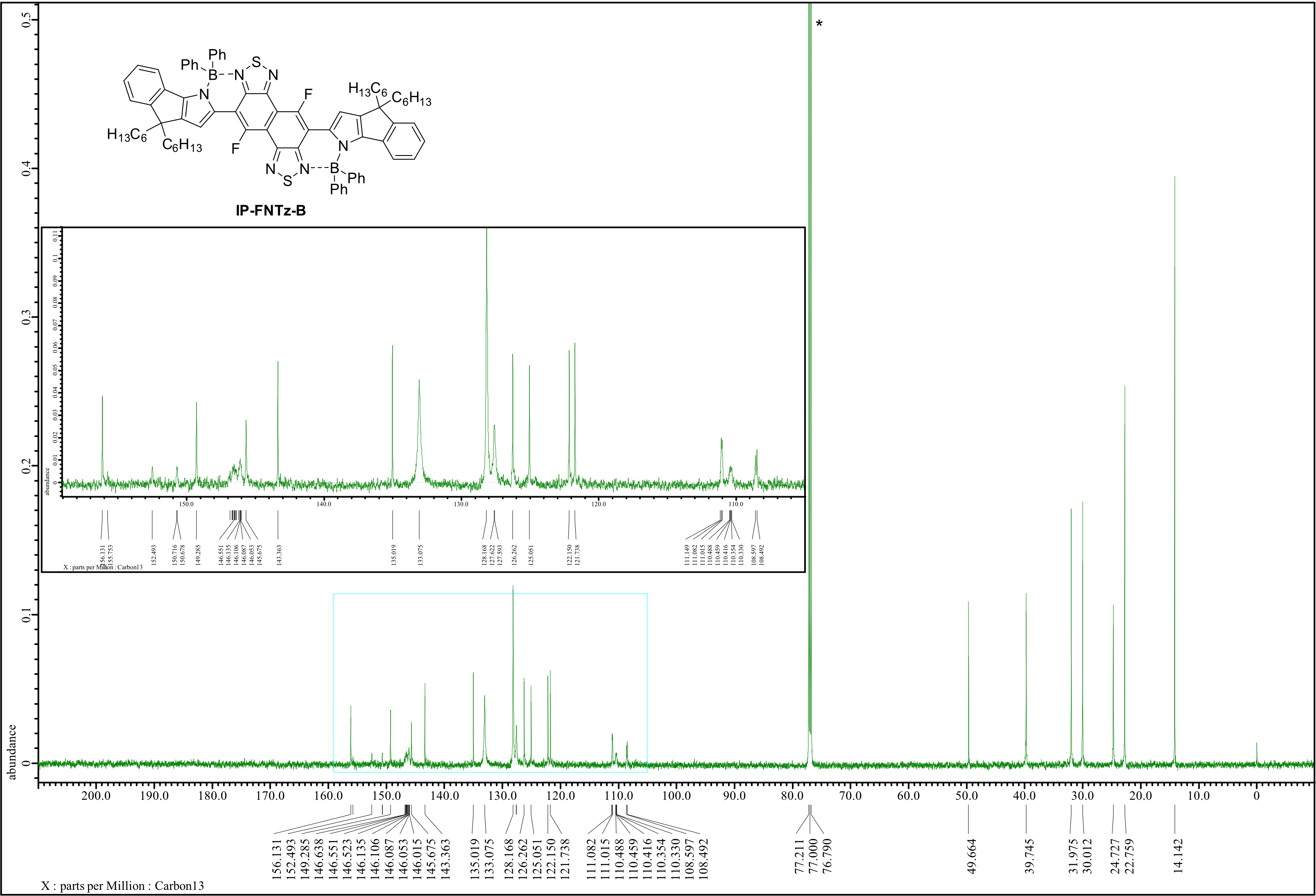


**Figure S23**. ^13^C NMR (151 MHz, CDCl_3_, r.t.) spectrum of **IP-FNTz-B**. Asterisk denotes solvent peaks.

**Computational Details and Data**

All calculations were conducted using the Gaussian 9 program.^[S9]^ The molecular geometries were optimized with the Becke Hybrid (B3LYP) at the 6-31G(d,p) level. Time-dependent density functional theory (TD-DFT) calculations were conducted at the CAM-B3LYP/6-31G(d,p) level of theory. The TD-DFT data were analyzed and summarized using GaussSum 3.0.^[S10]^ To estimate the biradical character (*y*), the occupation number of the lowest unoccupied natural orbital (LUNO) and the highest occupied natural orbital (HONO) for **Py-FNTz’, Py-FNTz-B’, IP-FNTz’, and IP-FNTz-B’** were calculated using the spin-unrestricted Hartree−Fock method at the UHF/6-31G(d) level.^[S11]^ Charge transfer integrals in the crystal packing structures for **IP-FNTz** and **IP-FNTz-B** were calculated using the ADF2022 program^[S12]^ at the PW91/TZP level.

**Table S3**. Calculated excited states of the optimized structure **Py-FNTz’** at the TD-CAM-B3LYP/6-31g(d,p).

| No. | Wavelength [nm] | Oscillator Strength | Major contributions (≥ 10%) | Minor contributions |
| --- | --- | --- | --- | --- |
| 1 | 552.5 | 0.5297 | HOMO->LUMO (98%) |  |
| 2 | 390.5 | 0.0000 | H-1->LUMO (70%), HOMO->L+1 (29%) |  |
| 3 | 360.5 | 0.0000 | H-1->LUMO (28%), HOMO->L+1 (68%) |  |
| 4 | 318.8 | 0.1952 | H-4->LUMO (52%), HOMO->L+2 (29%) | H-2->LUMO (5%), H-1->L+1 (5%),  HOMO->L+3 (5%) |
| 5 | 292.8 | 0.1076 | H-2->LUMO (81%), HOMO->L+2 (12%) | H-3->L+1 (4%) |
| 6 | 292.6 | 0.0000 | H-3->LUMO (93%) | H-2->L+1 (4%) |
| 7 | 289.1 | 0.9032 | H-4->LUMO (23%), HOMO->L+2 (53%) | H-2->LUMO (7%), H-1->L+1 (6%),  H-1->L+6 (2%), HOMO->L+3 (6%) |
| 8 | 275.9 | 0.0533 | H-5->LUMO (37%),  H-1->L+1 (52%) | H-4->LUMO (6%) |
| 9 | 267.4 | 0.0823 | H-5->LUMO (54%),  H-1->L+1 (26%) | H-6->L+1 (4%), H-4->LUMO (9%),  HOMO->L+3 (3%) |
| 10 | 265.0 | 0.0000 | H-6->LUMO (69%),  H-4->L+1 (13%) | H-1->L+2 (8%), HOMO->L+6 (4%) |

**Table S4**. Calculated excited states of the optimized structure **Py-FNTz-B’** at the TD-CAM-B3LYP/6-31g(d,p).

| No. | Wavelength [nm] | Oscillator Strength | Major contributions (≥ 10%) | Minor contributions |
| --- | --- | --- | --- | --- |
| 1 | 805.9 | 0.4167 | HOMO->LUMO (97%) |  |
| 2 | 536.8 | 0.0000 | H-1->LUMO (30%),  HOMO->L+1 (68%) |  |
| 3 | 468.6 | 0.0000 | H-1->LUMO (67%),  HOMO->L+1 (29%) |  |
| 4 | 402.9 | 0.0282 | H-2->LUMO (75%) | H-10->LUMO (3%), H-8->LUMO (4%),  H-4->LUMO (5%), H-3->L+1 (7%) |
| 5 | 400.8 | 0.0000 | H-3->LUMO (77%) | H-11->LUMO (5%), H-9->LUMO (3%),  H-5->LUMO (4%), H-2->L+1 (7%) |
| 6 | 382.7 | 0.0138 | H-5->L+1 (10%),  H-4->LUMO (68%) | H-6->LUMO (7%), H-2->LUMO (8%) |
| 7 | 382.1 | 0.0000 | H-5->LUMO (70%),  H-4->L+1 (10%) | H-7->LUMO (7%), H-3->LUMO (7%) |
| 8 | 362.1 | 0.0326 | H-8->LUMO (16%),  H-1->L+1 (43%) | H-13->LUMO (3%), H-12->LUMO (7%), H-10->LUMO (8%), H-6->LUMO (3%), HOMO->L+2 (7%), HOMO->L+4 (5%) |
| 9 | 360.3 | 0.0102 | H-6->LUMO (72%), H-4->LUMO (10%) | H-10->LUMO (6%), H-7->L+1 (7%) |
| 10 | 359.0 | 0.0000 | H-11->LUMO (12%),  H-7->LUMO (65%),  H-5->LUMO (11%) | H-6->L+1 (6%) |

**Table S5**. Calculated excited states of the optimized structure **IP-FNTz’** at the TD-CAM-B3LYP/6-31g(d,p).

| No. | Wavelength [nm] | Oscillator Strength | Major contributions (≥ 10%) | Minor contributions |
| --- | --- | --- | --- | --- |
| 1 | 652.5 | 1.0177 | HOMO->LUMO (97%) |  |
| 2 | 454.2 | 0.0000 | H-1->LUMO (88%) | HOMO->L+1 (9%) |
| 3 | 397.7 | 0.0000 | HOMO->L+1 (86%) | H-2->L+1 (3%), H-1->LUMO (9%) |
| 4 | 344.5 | 1.0353 | HOMO->L+2 (77%) | H-7->LUMO (6%), H-2->LUMO (5%), H-1->L+3 (4%) |
| 5 | 320.4 | 0.5490 | H-7->LUMO (12%), H-2->LUMO (22%), H-1->L+1 (36%), HOMO->L+2 (11%), HOMO->L+4 (10%) | H-6->LUMO (2%), H-1->L+3 (3%) |
| 6 | 305.7 | 0.0305 | H-2->LUMO (61%), H-1->L+1 (14%) | H-9->LUMO (3%), H-7->LUMO (6%), H-6->LUMO (3%), HOMO->L+4 (3%) |
| 7 | 297.5 | 0.2706 | H-7->LUMO (49%), H-1->L+1 (38%) | HOMO->L+4 (3%) |
| 8 | 297.3 | 0.0000 | H-5->LUMO (53%), H-3->LUMO (33%) | H-2->L+1 (2%), H-1->L+2 (3%) |
| 9 | 291.7 | 0.0000 | H-1->L+2 (30%), HOMO->L+3 (47%) | H-10->LUMO (4%), H-5->LUMO (2%), H-3->LUMO (4%), H-1->L+4 (2%) |
| 10 | 288.3 | 0.0256 | H-6->LUMO (51%), H-4->LUMO (31%) | H-9->LUMO (5%), H-7->LUMO (3%), H-1->L+1 (3%) |

**Table S6**. Calculated excited states of the optimized structure **IP-FNTz-B’** at the TD-CAM-B3LYP/6-31g(d,p).

| No. | Wavelength [nm] | Oscillator Strength | Major contributions (≥ 10%) | Minor contributions |
| --- | --- | --- | --- | --- |
| 1 | 1005.4 | 0.6678 | HOMO->LUMO (97%) |  |
| 2 | 621.7 | 0.0006 | H-1->LUMO (49%),  HOMO->L+1 (48%) |  |
| 3 | 549.8 | 0.0022 | H-1->LUMO (48%),  HOMO->L+1 (48%) |  |
| 4 | 403.8 | 0.0687 | H-1->L+1 (72%) | H-10->LUMO (3%), H-4->LUMO (5%), H-2->LUMO (4%), HOMO->L+3 (2%), HOMO->L+4 (2%) |
| 5 | 392.5 | 0.0011 | H-9->LUMO (10%), H-5->LUMO (11%), H-3->LUMO (56%) | H-13->LUMO (3%), H-11->LUMO (2%), H-7->LUMO (8%), H-2->L+1 (5%) |
| 6 | 392.1 | 0.0506 | H-4->LUMO (11%), H-2->LUMO (44%), H-1->L+1 (11%) | H-12->LUMO (2%), H-10->LUMO (4%), H-8->LUMO (7%), H-6->LUMO (8%), H-3->L+1 (4%) |
| 7 | 387.2 | 1.4909 | HOMO->L+2 (84%) | H-2->LUMO (3%), H-1->L+6 (4%) |
| 8 | 378.8 | 0.0135 | H-4->LUMO (56%),  H-2->LUMO (25%) | H-8->LUMO (5%), H-5->L+1 (7%), H-3->L+1 (3%) |
| 9 | 376.7 | 0.0143 | H-5->LUMO (65%),  H-3->LUMO (18%) | H-9->LUMO (4%), H-4->L+1 (6%), H-2->L+1 (3%) |
| 10 | 358.3 | 0.0018 | H-12->LUMO (18%),  H-10->LUMO (34%), H-8->LUMO (29%) | H-17->LUMO (2%), H-13->L+1 (4%), H-11->L+1 (3%), H-6->LUMO (2%) |

**Table S7**. Calculated excited states of the optimized structure **L2B2** at the TD-CAM-B3LYP/6-31g(d,p).

| No. | Wavelength [nm] | Oscillator Strength | Major contributions (≥ 10%) | Minor contributions |
| --- | --- | --- | --- | --- |
| 1 | 760.6 | 0.2418 | HOMO->LUMO (95%) | H-2->L+1 (4%) |
| 2 | 698.1 | 0.0000 | HOMO->L+1 (94%) | H-2->LUMO (4%) |
| 3 | 568.8 | 0.3232 | H-1->LUMO (93%) | H-11->L+1 (3%), H-2->L+1 (2%) |
| 4 | 529.5 | 0.0000 | H-1->L+1 (91%) | H-11->LUMO (4%), H-2->LUMO (2%) |
| 5 | 375.0 | 0.0000 | H-3->L+1 (15%), H-2->LUMO (71%) | H-12->L+1 (2%), H-4->LUMO (3%), HOMO->L+1 (3%) |
| 6 | 368.3 | 0.1316 | H-3->LUMO (31%), H-2->L+1 (37%), HOMO->L+2 (15%) | H-4->L+1 (9%) |
| 7 | 360.1 | 0.2392 | H-4->L+1 (22%), H-3->LUMO (13%), HOMO->L+2 (47%) | H-5->LUMO (9%) |
| 8 | 358.9 | 0.0000 | H-5->L+1 (15%), H-4->LUMO (51%), H-3->L+1 (16%) | H-7->L+1 (6%), H-2->LUMO (5%) |
| 9 | 352.5 | 0.1337 | H-8->L+1 (11%), H-7->LUMO (15%), H-5->LUMO (20%), H-2->L+1 (12%), HOMO->L+2 (26%) | H-4->L+1 (8%) |
| 10 | 342.8 | 0.0000 | H-8->LUMO (27%), H-6->LUMO (22%), H-5->L+1 (21%), H-3->L+1 (12%) | H-12->L+1 (5%), H-2->LUMO (2%) |

**Cartesian coordinates [Å] of the optimized structure of compound Py-FNTz’ at the ground state (S_0_)**1 C -1.553553 1.071300 -0.000047

2 C -0.181560 0.683081 -0.000114

3 C 0.181559 -0.683083 -0.000112

4 C -0.902561 -1.636918 -0.000137

5 C -2.286674 -1.184748 -0.000077

6 C -2.642221 0.215663 0.000015

7 C 0.902560 1.636915 -0.000142

8 C 2.286673 1.184745 -0.000081

9 C 2.642220 -0.215666 0.000015

10 N -0.780867 -2.964257 -0.000209

11 S -2.293948 -3.595200 -0.000336

12 N -3.163067 -2.191938 -0.000108

13 N 0.780866 2.964255 -0.000232

14 S 2.293949 3.595196 -0.000312

15 N 3.163068 2.191934 -0.000129

16 C 1.553551 -1.071302 -0.000052

17 C 4.015962 -0.678719 0.000150

18 C 4.571523 -1.965177 0.000392

19 N 5.079264 0.208008 0.000094

20 C 6.258809 -0.472026 0.000286

21 C 5.974899 -1.828399 0.000476

22 C -4.015962 0.678719 0.000151

23 N -5.079267 -0.208003 0.000121

24 C -6.258810 0.472036 0.000299

25 C -5.974894 1.828408 0.000456

26 C -4.571517 1.965180 0.000364

27 F -1.795912 2.393152 -0.000001

28 F 1.795909 -2.393155 -0.000016

29 H 4.015806 -2.887671 0.000497

30 H 4.948263 1.211807 -0.000056

31 H 7.203710 0.051002 0.000275

32 H 6.701564 -2.628081 0.000654

33 H -4.948271 -1.211802 -0.000019

34 H -7.203713 -0.050988 0.000304

35 H -6.701555 2.628093 0.000620

36 H -4.015796 2.887671 0.000445

B3LYP/6-31g(d,p) opt

E = -2015.28825563 hartree

B3LYP/6-31g(d,p) opt

# of imaginary frequencies = 0

**Cartesian coordinates [Å] of the optimized structure of Py-FNTz-B’ at the ground state (S_0_)**1 C 0.952857 0.926987 1.346079

2 C -0.117569 0.433453 0.553746

3 C 0.1177 -0.43403 -0.553599

4 C 1.499276 -0.757435 -0.821964

5 C 2.534303 -0.19259 0.008606

6 C 2.295938 0.647909 1.134865

7 C -1.499131 0.756939 0.822055

8 C -2.534195 0.192011 -0.008427

9 C -2.295844 -0.648743 -1.134522

10 C -0.952754 -0.927762 -1.345773

11 N 1.974521 -1.549371 -1.783495

12 S 3.601265 -1.608342 -1.689758

13 N 3.756441 -0.567549 -0.377339

14 N -1.974329 1.549092 1.783421

15 S -3.601071 1.608195 1.689632

16 N -3.756298 0.5672 0.377365

17 C 3.409179 1.035851 1.950113

18 C -3.409101 -1.036963 -1.949593

19 F -0.633973 -1.70753 -2.390709

20 F 0.634067 1.706635 2.3911

21 N 4.694096 0.643482 1.577758

22 C 5.543222 1.044767 2.562096

23 C 4.831637 1.692493 3.570084

24 C 3.481227 1.68887 3.188324

25 N -4.694037 -0.644625 -1.577287

26 C -5.543201 -1.046547 -2.561335

27 C -4.831627 -1.694668 -3.569067

28 C -3.481181 -1.690648 -3.187459

29 B 5.149432 0.102369 0.186535

30 B -5.149491 -0.102412 -0.186477

31 C -5.497313 -1.30408 0.853571

32 C 6.257532 -1.067175 0.332918

33 C 5.496478 1.304845 -0.852862

34 C 5.928158 -2.293411 0.944496

35 C 6.87113 -3.30452 1.125751

36 C 8.188888 -3.110798 0.705637

37 C 8.54794 -1.902124 0.109951

38 C 7.592601 -0.898852 -0.073297

39 C 5.917741 1.038558 -2.169414

40 C 6.193393 2.059954 -3.078827

41 C 6.055905 3.394159 -2.690656

42 C 5.644231 3.688315 -1.39136

43 C 5.367259 2.656635 -0.490618

44 C -6.256966 1.067576 -0.333975

45 C -5.367535 -2.65616 0.492591

46 C -5.644981 -3.687118 1.394008

47 C -6.05772 -3.391932 2.692735

48 C -6.195776 -2.057425 3.079655

49 C -5.919626 -1.036755 2.169581

50 C -7.592447 0.899809 0.071162

51 C -8.547299 1.903334 -0.113193

52 C -8.187337 3.111719 -0.70894

53 C -6.869166 3.304871 -1.127999

54 C -5.926678 2.293504 -0.945639

55 H 6.602239 0.848052 2.48115

56 H 5.250879 2.102394 4.477985

57 H 2.639943 2.077822 3.740616

58 H -6.602233 -0.849903 -2.480381

59 H -5.250903 -2.105115 -4.476706

60 H -2.639898 -2.079719 -3.739665

61 H 4.912284 -2.457685 1.298693

62 H 6.58172 -4.239377 1.598366

63 H 8.928413 -3.894409 0.845363

64 H 9.572017 -1.7389 -0.215097

65 H 7.891017 0.033883 -0.542521

66 H 6.051765 0.008378 -2.494559

67 H 6.519458 1.815701 -4.086396

68 H 6.269884 4.194222 -3.393844

69 H 5.536019 4.722755 -1.076007

70 H 5.049627 2.912048 0.516033

71 H -5.049117 -2.912373 -0.513613

72 H -5.536341 -4.721809 1.079627

73 H -6.272078 -4.191443 3.396434

74 H -6.522658 -1.81237 4.086765

75 H -6.054068 -0.006318 2.49374

76 H -7.891553 -0.032687 0.540418

77 H -9.5717 1.740569 0.211064

78 H -8.926491 3.895523 -0.849545

79 H -6.57904 4.239483 -1.600659

80 H -4.910458 2.45734 -1.299043

B3LYP/6-31g(d,p) opt

E = -2990.52902177 hartree

B3LYP/6-31g(d,p) freq

# of imaginary frequencies = 0

**Cartesian coordinates [Å] of the optimized structure of IP-FNTz’ at the ground state (S_0_)**1 C -1.677353 0.859871 -0.000568

2 C -0.270177 0.654749 -0.000672

3 C 0.270181 -0.654701 -0.000669

4 C -0.680963 -1.741642 -0.000743

5 C -2.112616 -1.474562 -0.000650

6 C -2.649150 -0.131473 -0.000457

7 C 0.680966 1.741691 -0.000748

8 C 2.112620 1.474612 -0.000655

9 C 2.649154 0.131522 -0.000458

10 N -0.386952 -3.041598 -0.001002

11 S -1.804190 -3.866641 -0.000865

12 N -2.848986 -2.587925 -0.000834

13 N 0.386953 3.041645 -0.001021

14 S 1.804187 3.866693 -0.000845

15 N 2.848985 2.587979 -0.000855

16 F -2.091153 2.139536 -0.000519

17 C 1.677357 -0.859821 -0.000569

18 F 2.091162 -2.139484 -0.000524

19 C 4.067776 -0.146153 -0.000211

20 C 4.782283 -1.365585 0.000344

21 N 5.003062 0.886485 -0.000397

22 C 6.248330 0.347389 0.000015

23 C 6.144892 -1.039869 0.000477

24 C -4.067775 0.146186 -0.000207

25 N -5.003040 -0.886471 -0.000391

26 C -6.248319 -0.347401 0.000025

27 C -6.144908 1.039859 0.000482

28 C -4.782306 1.365603 0.000345

29 C -7.629540 -0.778497 0.000171

30 C -8.407851 0.410699 0.000754

31 C -7.523478 1.675181 0.001004

32 C 7.629559 0.778457 0.000166

33 C 8.407848 -0.410755 0.000756

34 C 7.523449 -1.675219 0.001006

35 C -8.240123 -2.034411 -0.000137

36 C -9.636079 -2.101325 0.000145

37 C -10.406282 -0.936012 0.000720

38 C -9.791956 0.325436 0.001026

39 C 8.240168 2.034359 -0.000144

40 C 9.636125 2.101244 0.000144

41 C 10.406306 0.935917 0.000726

42 C 9.791954 -0.325519 0.001033

43 C -7.767432 2.527247 -1.263915

44 C -7.766871 2.526345 1.266640

45 C 7.766819 -2.526384 1.266646

46 C 7.767391 -2.527294 -1.263909

47 H 4.330284 -2.343242 0.000594

48 H 4.729341 1.861721 -0.000704

49 H -4.729299 -1.861702 -0.000685

50 H -4.330328 2.343269 0.000591

51 H -7.646580 -2.944278 -0.000586

52 H -10.126357 -3.070501 -0.000086

53 H -11.489943 -1.006491 0.000933

54 H -10.402667 1.224641 0.001473

55 H 7.646643 2.944238 -0.000598

56 H 10.126423 3.070411 -0.000089

57 H 11.489967 1.006374 0.000942

58 H 10.402647 -1.224737 0.001485

59 H -7.083543 3.382287 -1.284763

60 H -8.791728 2.914464 -1.283370

61 H -7.605646 1.938756 -2.171153

62 H -7.604674 1.937209 2.173385

63 H -7.082979 3.381375 1.287791

64 H -8.791161 2.913540 1.286829

65 H 7.082913 -3.381403 1.287795

66 H 8.791103 -2.913595 1.286842

67 H 7.604626 -1.937242 2.173389

68 H 7.083485 -3.382319 -1.284759

69 H 7.605622 -1.938802 -2.171150

70 H 8.791679 -2.914532 -1.283358

B3LYP/6-31g(d,p) opt

E = -2710.89750964 hartree

B3LYP/6-31g(d,p) freq

# of imaginary frequencies = 0

**Cartesian coordinates [Å] of the optimized structure of IP-FNTz-B’ at the ground state (S_0_)**1 C -1.360386 -1.311719 0.090669

2 C -0.078800 -0.711104 0.069170

3 C 0.078759 0.711114 0.069125

4 C -1.143379 1.480208 0.063407

5 C -2.412744 0.793152 0.034956

6 C -2.569794 -0.620225 0.089076

7 C 1.143336 -1.480195 0.063480

8 C 2.412695 -0.793138 0.034951

9 C 2.569751 0.620239 0.088981

10 C 1.360341 1.311734 0.090579

11 N -1.269549 2.806351 0.082515

12 S -2.850851 3.209377 0.067124

13 N -3.446168 1.632720 0.001699

14 N 1.269508 -2.806341 0.082694

15 S 2.850816 -3.209348 0.067341

16 N 3.446124 -1.632703 0.001726

17 F -1.405921 -2.653910 0.136320

18 F 1.405882 2.653926 0.136167

19 C -3.887365 -1.162738 0.212189

20 N -5.009030 -0.314028 0.192766

21 C -6.090909 -1.125376 0.401653

22 C -5.679023 -2.455580 0.571655

23 C -4.294911 -2.491977 0.449410

24 C -7.545724 -1.043625 0.465594

25 C -8.008210 -2.366354 0.712030

26 C -6.853858 -3.376573 0.809880

27 C -8.464206 0.001737 0.320507

28 C -9.828289 -0.274410 0.434412

29 C -10.281134 -1.571031 0.687455

30 C -9.366498 -2.623224 0.825664

31 C -6.964091 -4.464708 -0.280575

32 C -6.797855 -4.033158 2.207430

33 B -5.015595 1.199708 -0.224861

34 C -5.307988 1.378368 -1.818552

35 C -5.171929 2.617709 -2.474010

36 C -5.417559 2.769715 -3.839076

37 C -5.810813 1.670281 -4.603254

38 C -5.948455 0.428223 -3.985624

39 C -5.696333 0.289281 -2.618750

40 C -7.362791 3.992415 1.328459

41 C -6.697236 3.184820 0.401049

42 C -5.857915 2.131815 0.805368

43 C -5.721245 1.925355 2.192784

44 C -6.382563 2.722116 3.125909

45 C -7.207131 3.764037 2.694721

46 C 3.887323 1.162750 0.212082

47 N 5.008992 0.314043 0.192645

48 C 6.090871 1.125395 0.401515

49 C 5.678984 2.455599 0.571522

50 C 4.294871 2.491991 0.449296

51 C 7.545681 1.043632 0.465529

52 C 8.008165 2.366356 0.711997

53 C 6.853815 3.376582 0.809806

54 C 8.464160 -0.001738 0.320487

55 C 9.828240 0.274396 0.434468

56 C 10.281083 1.571013 0.687537

57 C 9.366450 2.623213 0.825702

58 C 6.797751 4.033149 2.207363

59 C 6.964105 4.464731 -0.280629

60 B 5.015552 -1.199731 -0.224863

61 C 5.307991 -1.378515 -1.818544

62 C 5.171761 -2.617830 -2.474009

63 C 5.417498 -2.769887 -3.839051

64 C 5.811034 -1.670537 -4.603202

65 C 5.948851 -0.428502 -3.985566

66 C 5.696621 -0.289508 -2.618718

67 C 6.382653 -2.721865 3.125986

68 C 5.721254 -1.925210 2.192826

69 C 5.857891 -2.131764 0.805421

70 C 6.697258 -3.184750 0.401147

71 C 7.362893 -3.992237 1.328591

72 C 7.207273 -3.763763 2.694842

73 H -3.627588 -3.335329 0.532661

74 H -8.128633 1.011820 0.127736

75 H -10.544715 0.534389 0.323901

76 H -11.346083 -1.766188 0.773550

77 H -9.723484 -3.632175 1.015566

78 H -6.094987 -5.130243 -0.245768

79 H -7.861493 -5.075051 -0.134265

80 H -7.012762 -4.018210 -1.277554

81 H -6.723034 -3.276758 2.993484

82 H -7.695338 -4.632729 2.392889

83 H -5.928500 -4.694771 2.284675

84 H -4.866779 3.498976 -1.913897

85 H -5.300554 3.744806 -4.304474

86 H -6.003100 1.781166 -5.666711

87 H -6.248823 -0.438950 -4.567737

88 H -5.804355 -0.692718 -2.169641

89 H -8.006980 4.795563 0.980420

90 H -6.850434 3.369233 -0.657781

91 H -5.089695 1.114187 2.548190

92 H -6.259086 2.529966 4.188415

93 H -7.725459 4.387393 3.417999

94 H 3.627547 3.335343 0.532542

95 H 8.128589 -1.011821 0.127705

96 H 10.544664 -0.534409 0.323990

97 H 11.346030 1.766161 0.773678

98 H 9.723433 3.632162 1.015627

99 H 5.928391 4.694759 2.284576

100 H 7.695225 4.632721 2.392865

101 H 6.722900 3.276739 2.993403

102 H 7.012806 4.018246 -1.277613

103 H 6.095008 5.130278 -0.245848

104 H 7.861510 5.075060 -0.134278

105 H 4.866398 -3.499046 -1.913933

106 H 5.300347 -3.744960 -4.304452

107 H 6.003396 -1.781469 -5.666639

108 H 6.249441 0.438612 -4.567653

109 H 5.804781 0.692475 -2.169607

110 H 6.259193 -2.529659 4.188484

111 H 5.089661 -1.114059 2.548197

112 H 6.850401 -3.369237 -0.657676

113 H 8.007121 -4.795371 0.980592

114 H 7.725681 -4.387020 3.418148

B3LYP/6-31g(d,p) opt

E = -3686.13232328 hartree

B3LYP/6-31g(d,p) freq

# of imaginary frequencies = 0

**Cartesian coordinates [Å] of the optimized structure of compound L2B2 at the ground state (S_0_)**1 C -2.825274 -1.730088 -0.182059

2 C 4.746949 -1.376595 -4.418509

3 C -1.396009 -0.014872 -0.066156

4 H 6.421302 -2.555381 -3.739175

5 C -0.779739 1.227398 0.022002

6 H 3.011893 -0.147133 -4.766941

7 C 0.623933 1.238671 0.078911

8 H 4.947388 -1.543754 -5.473241

9 C 1.396011 0.014876 0.066150

10 C 3.844271 -1.962612 0.887819

11 C 0.779741 -1.227394 -0.022008

12 C 3.879503 -3.287269 0.419589

13 C -0.623930 -1.238667 -0.078917

14 C 3.717495 -1.784536 2.279985

15 H -1.343845 2.150963 0.032664

16 C 3.800990 -4.377659 1.291118

17 H 1.343848 -2.150959 -0.032669

18 H 3.960492 -3.472222 -0.647254

19 C 2.825277 1.730092 0.182057

20 C 3.641547 -2.863712 3.159104

21 N -2.748085 -0.335768 -0.140212

22 H 3.663633 -0.774934 2.682300

23 N 2.748087 0.335772 0.140205

24 C 3.685350 -4.169591 2.664865

25 C -4.102308 -2.382409 -0.331414

26 H 3.829504 -5.389224 0.894391

27 C -5.249278 -1.551902 -0.507150

28 H 3.542334 -2.688774 4.227221

29 C -4.352878 -3.747194 -0.393964

30 H 3.625234 -5.014842 3.345035

31 C -6.563729 -2.067956 -0.797708

32 C -4.221838 0.937765 1.660373

33 C -5.652134 -4.268054 -0.646681

34 C -5.307579 1.722658 2.093658

35 H -3.531769 -4.443944 -0.264570

36 C -3.411281 0.378826 2.663617

37 C -6.757482 -3.473311 -0.861926

38 C -5.572945 1.942456 3.445787

39 H -5.765295 -5.347335 -0.684022

40 H -5.965543 2.186911 1.361319

41 H -7.738590 -3.880528 -1.072202

42 C -3.663342 0.594458 4.020726

43 C 4.102311 2.382411 0.331417

44 H -2.562043 -0.235454 2.381642

45 C 5.249281 1.551902 0.507150

46 C -4.746939 1.376582 4.418510

47 C 4.352882 3.747195 0.393972

48 H -6.421331 2.555318 3.739186

49 C 6.563732 2.067954 0.797709

50 H -3.011845 0.147171 4.766933

51 C 5.652138 4.268054 0.646691

52 H -4.947374 1.543739 5.473243

53 H 3.531774 4.443947 0.264582

54 C -3.844277 1.962616 -0.887818

55 C 6.757487 3.473309 0.861932

56 C -3.879508 3.287272 -0.419583

57 H 5.765301 5.347334 0.684037

58 C -3.717511 1.784545 -2.279985

59 H 7.738595 3.880524 1.072210

60 C -3.801002 4.377664 -1.291109

61 N -5.220623 -0.220180 -0.449962

62 H -3.960490 3.472220 0.647261

63 N -7.478669 -1.103087 -0.976316

64 C -3.641569 2.863724 -3.159101

65 N 5.220625 0.220180 0.449957

66 H -3.663650 0.774945 -2.682304

67 N 7.478671 1.103084 0.976314

68 C -3.685370 4.169602 -2.664858

69 B -3.927743 0.660761 0.080871

70 H -3.829514 5.389228 -0.894379

71 B 3.927744 -0.660761 -0.080874

72 H -3.542364 2.688790 -4.227220

73 C 4.221840 -0.937770 -1.660374

74 H -3.625261 5.014855 -3.345025

75 C 5.307562 -1.722693 -2.093653

76 S 6.762106 -0.351400 0.794974

77 C 3.411306 -0.378806 -2.663622

78 S -6.762104 0.351398 -0.794982

79 C 5.572932 -1.942495 -3.445781

80 C 1.559788 2.299036 0.153913

81 H 5.965506 -2.186967 -1.361310

82 H 1.335279 3.354785 0.212495

83 C 3.663371 -0.594441 -4.020731

84 C -1.559785 -2.299032 -0.153917

85 H 2.562083 0.235498 -2.381652

86 H -1.335275 -3.354781 -0.212497

B3LYP/6-31g(d,p) opt

E = -2945.70996402 hartree

B3LYP/6-31g(d,p) freq

# of imaginary frequencies = 0

**References**

[S1] A. S. Klein, A. Domröse, P. Bongen, H. U. C. Brass, T. Classen, A. Loeschcke, T. Drepper, L. Laraia, S. Sievers, K.-E. Jaeger, J. Pietruszka, *ACS Synth. Biol.* **2017**, *6*, 1757–1765

[S2] C. W. Williams, R. Shenje, S. France, *J. Org. Chem.* **2016**, *81*, 8253–8267.

[S3] S. D. Dimitrov and J. R. Durrant, *Chem. Mater.*, **2014**, *26*, 616.

[S4] G. G. Malliaras, J. R. Salem, P. J. Brock and C. Scott, *Phys. Rev. B*, **1998**, *58*, 13411

[S5] C. Goh, R. J. Kline, M. D. McGehee, E. N. Kadnikova and J. M. J. Fréchet, *Appl. Phys. Lett.*, **2005**, *86*, 122110.

[S6] H. Puschmann, *J. Appl. Crystallogr.* **2009**, *42*, 339−341.

[S7] G. M. Sheldrick, *Acta Crystallogr., Sect. A: Found. Adv.* **2015**, *A71*, 3−8.

[S8] G. M. Sheldrick, *Acta Crystallogr., Sect. C: Struct. Chem.* **2015**, *C71*, 3− 8.

[S9] Gaussian 09, Revision D.01, M. J. Frisch, G. W. Trucks, H. B. Schlegel, G. E. Scuseria, M. A. Robb, J. R. Cheeseman, G. Scalmani, V. Barone, B. Mennucci, G. A. Petersson, H. Nakatsuji, M. Caricato, X. Li, H. P. Hratchian, A. F. Izmaylov, J. Bloino, G. Zheng, J. L. Sonnenberg, M. Hada, M. Ehara, K. Toyota, R. Fukuda, J. Hasegawa, M. Ishida, T. Nakajima, Y. Honda, O. Kitao, H. Nakai, T. Vreven, J. A. Montgomery, Jr., J. E. Peralta, F. Ogliaro, M. Bearpark, J. J. Heyd, E. Brothers, K. N. Kudin, V. N. Staroverov, T. Keith, R. Kobayashi, J. Normand, K. Raghavachari, A. Rendell, J. C. Burant, S. S. Iyengar, J. Tomasi, M. Cossi, N. Rega, J. M. Millam, M. Klene, J. E. Knox, J. B. Cross, V. Bakken, C. Adamo, J. Jaramillo, R. Gomperts, R. E. Stratmann, O. Yazyev, A. J. Austin, R. Cammi, C. Pomelli, J. W. Ochterski, R. L. Martin, K. Morokuma, V. G. Zakrzewski, G. A. Voth, P. Salvador, J. J. Dannenberg, S. Dapprich, A. D. Daniels, O. Farkas, J. B. Foresman, J. V. Ortiz, J. Cioslowski, and D. J. Fox, Gaussian, Inc., Wallingford CT, 2013.

[S10] GaussSum: N. M. O'Boyle, A. L. Tenderholt and K. M. Langner. *J. Comp. Chem.* **2008**, *29*, 839–845.

[S11] R. Kishi, S. Ochi, S. Izumi, A. Makino, T. Nagami, J.-y. Fujiyoshi, N. Matsushita, M. Saito, M. Nakano, *Chem. Eur. J.* **2016**, *22*, 1493–1500.

[S12] ADF2022, SCM, Theoretical Chemistry. Vrije Universiteit, Amsterdam, The Netherlands, http://www.scm.com.
